# The SHAPES Smart Mirror Approach for Independent Living, Healthy and Active Ageing

**DOI:** 10.3390/s21237938

**Published:** 2021-11-28

**Authors:** Javier Dorado Chaparro, Jesus Fernandez-Bermejo Ruiz, Maria J. Santofimia Romero, Cristina Bolaños Peño, Luis Unzueta Irurtia, Meritxell Garcia Perea, Xavier del Toro Garcia, Felix J. Villanueva Molina, Sonja Grigoleit, Juan C. Lopez

**Affiliations:** 1 Computer Architecture and Networks Group, School of Computer Science, University of Castilla-La Mancha, 13071 Ciudad Real, Spain; jesus.fruiz@uclm.es (J.F.-B.R.); mariajose.santofimia@uclm.es (M.J.S.R.); cristina.bolanos@uclm.es (C.B.P.); xavier.deltoro@uclm.es (X.d.T.G.); felix.villanueva@uclm.es (F.J.V.M.); juancarlos.lopez@uclm.es (J.C.L.); 2 Vicomtech, Basque Research and Technology Alliance (BRTA), 20009 Donostia, Spain; lunzueta@vicomtech.org (L.U.I.); mgarciap@vicomtech.org (M.G.P.); 3 Fraunhofer Institute for Technological Trend Analysis INT, 53864 Euskirchen, Germany; sonja.grigoleit@int.fraunhofer.de

**Keywords:** smart mirror, elderly, active ageing, user-centred design, internet of things, ambient assisted living

## Abstract

The benefits that technology can provide in terms of health and support for independent living are in many cases not enough to break the barriers that prevent older adults from accepting and embracing technology. This work proposes a hardware and software platform based on a smart mirror, which is equipped with a set of digital solutions whose main focus is to overcome older adults’ reluctance to use technology at home and wearable devices on the move. The system has been developed in the context of two use cases: the support of independent living for older individuals with neurodegenerative diseases and the promotion of physical rehabilitation activities at home. Aspects such as reliability, usability, consumption of computational resources, performance and accuracy of the proposed platform and digital solutions have been evaluated in the initial stages of the pilots within the SHAPES project, an EU-funded innovation action. It can be concluded that the SHAPES smart mirror has the potential to contribute as a technological breakthrough to overcome the barriers that prevent older adults from engaging in the use of assistive technologies.

## 1. Introduction

The ageing of the world’s population is bringing new challenges to modern societies due to its many implications [1]. Based on population pyramid forecasts and the increase in life expectancy or the global average age [2,3], in a few years, the world’s population will have more people over age 65 than ever before. Based on these forecasts, having to address the different needs associated with ageing poses important challenges [4]. It is therefore necessary to identify and provide technological support to the aspects that are key to ageing with a high quality of life (QoL). It is also necessary to raise awareness about the facts that determine healthy ageing [5].

Loneliness is one of the major problems faced by older adults. In Spain, for instance, there are more than two million people over 65 living alone [6]. Besides loneliness, a sedentary lifestyle is also a major health hazard, specially as people age. Avoiding a sedentary lifestyle, maintaining adequate physical and mental activity and a balanced diet are crucial for coping with age-related deterioration [7]. Similarly, health management and proper medication [8], together with good rest [9], have been identified as important factors to facilitate healthy ageing with high QoL.

Over the last decade, the Internet of Things (IoT) [10] and more specifically, the Ambient Assisted Living (AAL) [11] field has witnessed different technological innovations aimed at providing safe and interconnected environments intended to support and extend independent living. Smart homes [12,13], active ageing platforms [14] and smart devices [15] aim to address the different challenges of healthy ageing. However, while developers focus their design on older adults, there are numerous barriers overlooked leading to a lack of engagement and technology acceptance [16]. According to [17], there is a list of factors that affect the attitude and intention to use technologies supporting independent living. These personal and device-related factors comprise user expectancy, biophysical ageing restrictions, anxiety, the previous required knowledge, intrinsic motivation, personality and privacy concerns. In this sense, some important aspects should be considered, especially when designing interfaces for older adults [16]:

- **Physical condition:** It is common for older adults to suffer physical impairments, so in the design of the interaction, vision impairment, haptic deterioration and hearing loss have to be considered.
- **Computer literacy:** Older adults, in their majority, are unfamiliar with technology, so interaction with the interface and even with the use of the certain menus, buttons or other graphical user interface elements can be challenging. They have also a limited understanding of the processes and very frequently find that the interface controls are non-intuitive.
- **Cognitive condition:** Older adults also present different cognitive impairments, such as the reduction of the attentional control, visuospatial function and working memory. For this reason, the development of very intuitive interfaces is necessary to increase its usability for older adults.

To overlook such aspects causes older adults to be reluctant to use assistive technologies. It has been recently discovered that, despite the benefits that the use of such technology innovations can bring to older adult’s health, this cannot work alone as a motivator to overcome such limiting factors [17]. For this reason, special efforts have to be addressed to minimize those common barriers found by older adults, and to that end, there are guidelines and standards specifically devoted to address such limitations [16]:

- **Interface and control design:** Text size, colour, and font, use of intuitive control elements, having constant feedback on what is being done and having contextual help, constitute the basis for interface design.
- **Input controls:** Devices with touch screen are more intuitive for this age group; however, if there are physical limitations, voice commands might be more convenient to execute different actions, or even as text input. Another option is gaze-tracking devices, although this form of interaction is recommended primarily for users with severe motor impairment. Finally, using TV-like interfaces, such as a TV remote control, makes them comfortable when using applications that incorporate them.
- **Natural language:** Older users are often confused by long messages with many options. If we offer the user too many choices they may feel confused and overwhelmed in making the right choice. On the other hand, using simple descriptive language makes the interface more intuitive for them.
- **Cognitive Evaluation:** The employment of digital assistance and smart devices in older adults allows caregivers to monitor and support their cognitive state, facilitating the early detection of cognitive decline.

The SHAPES project (https://shapes2020.eu/ [accessed on 20 August 2021]) (Smart and Healthy Ageing through People Engaging in Supportive Systems) is a European-funded Innovation Action intended to promote long-term healthy and active ageing as well as to maintain a high-quality standard of life. In order to do so, SHAPES provides a set of digital solutions, deployed on an EU-standardised open platform, supporting the factors that determine a healthy and active ageing. The validation of both the open platform and the digital solutions have been devised through a pilot campaign, organised in seven different themes. This paper presents an innovative platform based on a smart mirror that will be tested in two of the pilot themes proposed in SHAPES (https://shapes2020.eu/about-shapes/pilots/ [accessed on 10 September 2021]), namely: Pilot Theme 5, which is dedicated to the care for older individuals with neurodegenerative diseases, and Pilot Theme 6, which is focused on providing physical rehabilitation at home.

Mirrors have fascinated humans since their appearance, and it is no wonder that fiction is full of stories in which mirrors do much more than reflect, for example, predicting the future or revealing unconsciousness. Not only are mirrors appealing for fiction, but they have also been extensively studied in psychology. Mirrors, and more specifically their reflections, help us build our sense of self [18]. The SHAPES smart mirror approach is inspired by the human fascination for mirrors and the role they can play in enabling self-awareness by envisioning a future in which a smart mirror and a virtual assistant will guide individuals towards improved self-awareness and self-management of health.

This work describes the main features of the SHAPES smart mirror as well as the different digital solutions devised for Pilot Themes 5 and 6, providing support for independent living to people with neurodegenerative diseases and physical rehabilitation at home. The platform has been evaluated from the point of view of its reliability, usability, computational resource consumption and performance of its hardware and software components. Section 3 describes the different elements, both hardware and software, that comprise the proposed solution, as well as the methodologies used within the context of the SHAPES project. SHAPES Pilot Themes 5 and 6 are employed for validation purposes and Section 4 provides the details of the different setups considered for validating the system and the different digital solutions. It also discusses the result obtained during the validation of the pilot setups. Finally, Section 5 presents the main conclusions drawn from this work.

## 2. Previous Works

The main contribution of this paper is the smart-mirror-based platform, devoted to host and support the execution and communication of a set of digital solutions for independent living, and active and healthy ageing.

The smart mirror idea, consisting in a computer system with a screen covered by a two-way mirror, that provides the mirror functionality combined with the information displayed by the screen, has recently attracted attention as a smart home component. Certain versions might also include touch screen or even gesture control through a camera, providing more sophisticated interfaces.

An extensive list of previous works using smart mirrors for various purposes and with different features is presented in Table 1. This table summarizes the most relevant contributions to date, along with an analysis of the their most significant characteristics that can be potentially used to support independent living and active and healthy ageing.

From the analysis of the previous works it can be seen that reviewed systems are mostly designed for a general public (only [41] is intended for older people). The purpose of all the listed systems can be classified into: generic virtual assistant and home automation solutions and physical and psychological health solutions. Some solution encompass both a general home assistant and home automation with healthcare solutions. In the context of the reviewed solutions, the smart mirror platform presented in this paper provides some novelty beyond the state of the art due to its particular features:

- It aims to provide a comprehensive ambient assisted living solution for older individuals seeking active and healthy ageing, and extending independent living.
- It has been developed in the context of the SHAPES project and the broader technological platform it provides. This has enabled the co-design of the solution with different stakeholders and partners with broad experience in the field.
- Special emphasis is made in the pilots and use cases described: care for older individuals with neurodegenerative diseases and physical rehabilitation at home.
- It incorporates a rich combination of digital solutions for the aforementioned purpose comprising: home sensors with wireless communications, smart gateway functionalities, videoconferencing calls, wearable devices and cameras for physical activity monitoring, sound system, voice assistant, notifications and calendar reminder.
- It provides the means for interaction between the end users and therapists and caregivers.

Table 2 provides a comparison of the previous related work, described in Table 1, with the SHAPES smart mirror platform and the services it provides in the context of active and healthy ageing and independent living.

## 3. Materials and Methods

**Figure 1:**
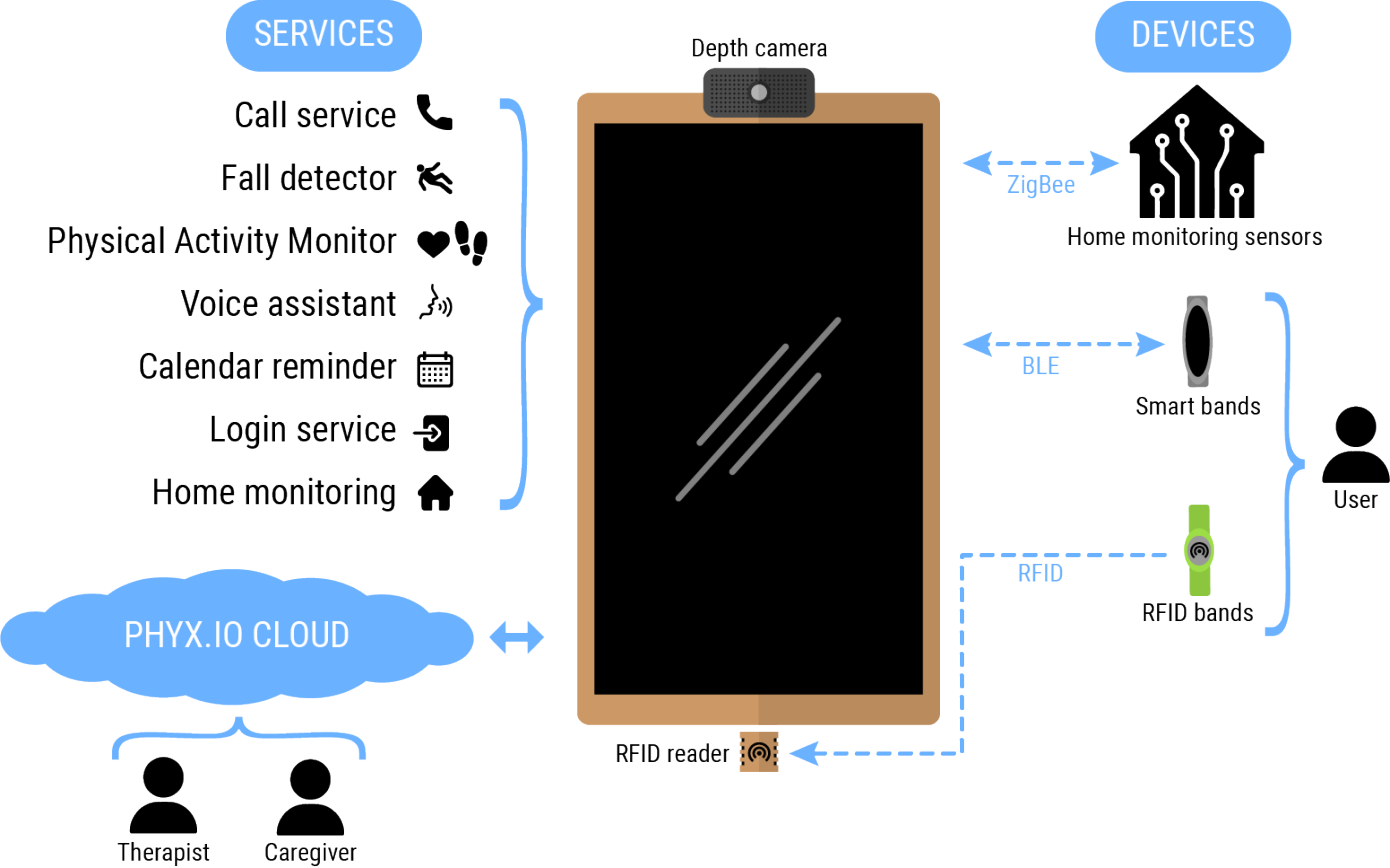
The SHAPES smart mirror ecosystem.

This paper presents an ecosystem consisting of cloud platforms, services and devices, developed fit-for-purpose to address the challenges faced by people suffering neurodegenerative diseases who wish to live independently, and for those that could benefit from having access to a personalized and at-home physical activity or rehabilitation assistance. Figure 1 (This diagram has been designed using resources from https://www.flaticon.com/ [accessed on 30 July 2021]) summarizes the main components of such ecosystem, having the smart mirror platform at its core. The smart mirror works as a computing node in which most of the applications are run. Furthermore, it also works as a smart gateway translating communication among different communication protocols and routing information between the cloud and the edge nodes. Table 3 summarizes the main services supported by the SHAPES smart mirror along with the different inputs and outputs being processed, as well as the addressed purpose of each one of them.

The following subsections describe in more detail the methodologies followed to develop the smart mirror within the SHAPES project framework, as well as the hardware components and services that comprise the proposed solution.

### 3.1. The SHAPES Digital Solutions Development Methodology

**Figure 2:**
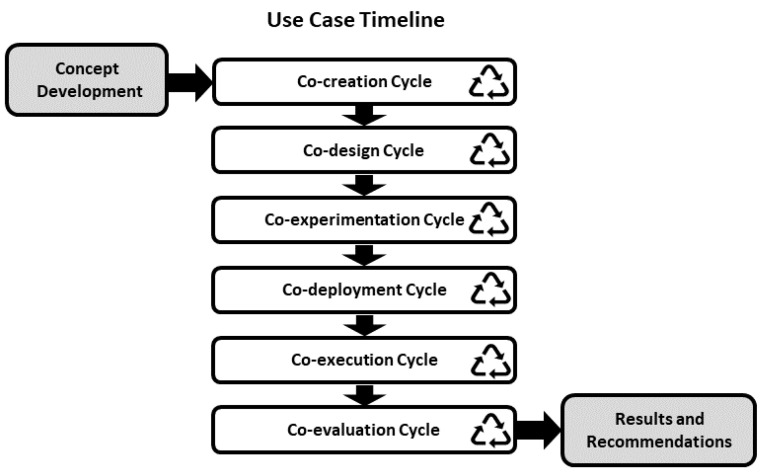
Use cases timeline defined in the SHAPES methodology.

The SHAPES project has put considerable efforts in engaging a relevant number of users and stakeholders so that they can participate in the co-creation, co-design and co-development of the platforms and the digital solutions that will be included in it. To this end, the SHAPES project has designed an incremental cycling methodology in which the different cycles are iterated, from the concept development towards the results and recommendations [42], as depicted in Figure 2. The SHAPES digital solution development methodology is based on the WHO guidelines described in the report “Monitoring and Evaluating Digital Health Interventions. A practical guide to conducting research and assessment” [43]. The digital solutions presented here have gone through this cycling methodology, in different iterations during which the use cases, the hardware (mainly the smart mirror) and the software interfaces and functionalities have been co-created, co-designed, co-experimented, co-deployed, co-executed and co-evaluated. The result and recommendation stage will follow after a long-run piloting during which the effectiveness of the interventions will be evaluated.

The SHAPES pilot campaign is organized into several pilots covering different themes (https://shapes2020.eu/about-shapes/pilots/ [accessed on 10 September 2021]). Each pilot theme is divide into several use cases that have been co-created and co-designed within the project consortium. Table 4 provides a detailed description of pilot campaign phases.

#### 3.1.1. The Co-Creation Cycle

During the co-creation cycle, different meetings were organized with relevant stakeholders including physical therapists, social workers, general practitioners, nurses, informal caregivers and older adults. These meetings were intended to address the following issues: (1) Identify the main challenges addressed by these different profiles in their endeavour to promote healthy and active ageing. (2) Identify main barriers in the use of commercial solutions relevant for the purpose of promoting healthy and active ageing. (3) Understand stakeholders’ main concerns regarding privacy and ethics.

Different use cases were co-created with the different stakeholders based on the issues that prompted major challenges and for which there was a lack of commercial solutions. During this stage, we also identified the different user personas that will mirror the target users of the different use cases. A user persona is a fictional character that is used to communicate in a more straight forward manner the key motivations, concerns and interest of a group of users, represented in the shape of that character [44]. Each of the identified personas will be described based on attributes, attitudes, behaviours and characteristics. The use case, on the other hand, are used to describe how these user personas will use the different digital solutions. The use case considers human–computer interaction and for that reason aspects such as usability or usefulness should be considered. The document in [45] summarizes the result of this stage, in which the co-created personas and use cases are described.

#### 3.1.2. The Co-Design Cycle

The co-design cycle has followed an agile approach based on SCRUM [46] during which different stakeholders have been involved playing the role of the Product Owner. This role holds several responsibilities such as holding the vision of what is expected from the digital solution as well as transmitting that vision to the software development team.

This stage of the development methodology has been supported in different artefacts or prototypes. In early stages, mock-ups were employed for evaluating aspects such as usability or usefulness. First, stakeholders were presented with interfaces developed in Pencil (https://pencil.evolus.vn/ [accessed on 15 September 2021]), a GUI prototyping tool, supporting navigation through the different menus, but without any real functionality. From the evaluation of the different Pencil projects, functional requirements were gathered and fed to the development team. As a result of the co-creation and co-design cycles the user requirements were defined for the physical activity monitoring with wearable devices use case (see Table 5) and the orofacial and physical rehabilitation use cases (see Table 6).

#### 3.1.3. The Co-Experimentation and Co-Deployment Cycle

These stages are intended to perform an early deployment of the different solutions so that errors or failures in meeting functional requirements can be early detected. First, during the co-experimentation stage the selected stakeholders are prompted to interact with the different solutions. During this stage the interaction is supervised and accompanied by the developers. The main idea is to observe the type of interaction the user has with the solution. This will provide very relevant qualitative information about the user perceptions.

The co-experimentation cycle therefore requires a host, with access to relevant stakeholders, in which different use cases can be simulated. The Nursing Home “El Salvador”, in Spain, was selected as the host for running the different cycles of the co-experimentation stage. This institution encompasses a relevant group of stakeholders (older adults, healthcare staff, caregivers, etc.).

After the co-experimentation cycle, the co-deployment cycle starts. Both stages are closely related and when no relevant missing requirements are detected from the co-experimentation, it is possible to continue straight away with the co-deployment stage. For the co-deployment cycle, the different digital solutions were tested during cycles of three to four weeks. Feedback was collected following informal interviews or formal questionnaires for usability and usefulness. Furthermore, data were collected and analyzed. Different errors or unexpected behaviours from the digital solutions were identified as results of having real users experimentation. This stage feeds the development stage with new requirements that have to be satisfied in new versions of the software.

#### 3.1.4. The Co-Execution Cycle

During this stage, the different digital solutions are deployed in real scenarios for extensive use in multiple sites. This will bring into light relevant structural societal issues not considered during previous stages of the development process.

#### 3.1.5. The Co-Evaluation Cycle

This stage performs the co-evaluation of the different solutions based on the list of key performance indicators (KPIs) and functional and non-functional requirements identified during the co-design stage. Moreover, usability and usefulness will also be evaluated in order to determine the user perception about the different tools as well as their willingness to use the devised technological solution.

### 3.2. The SHAPES Smart Mirror Hardware

**Figure 3:**
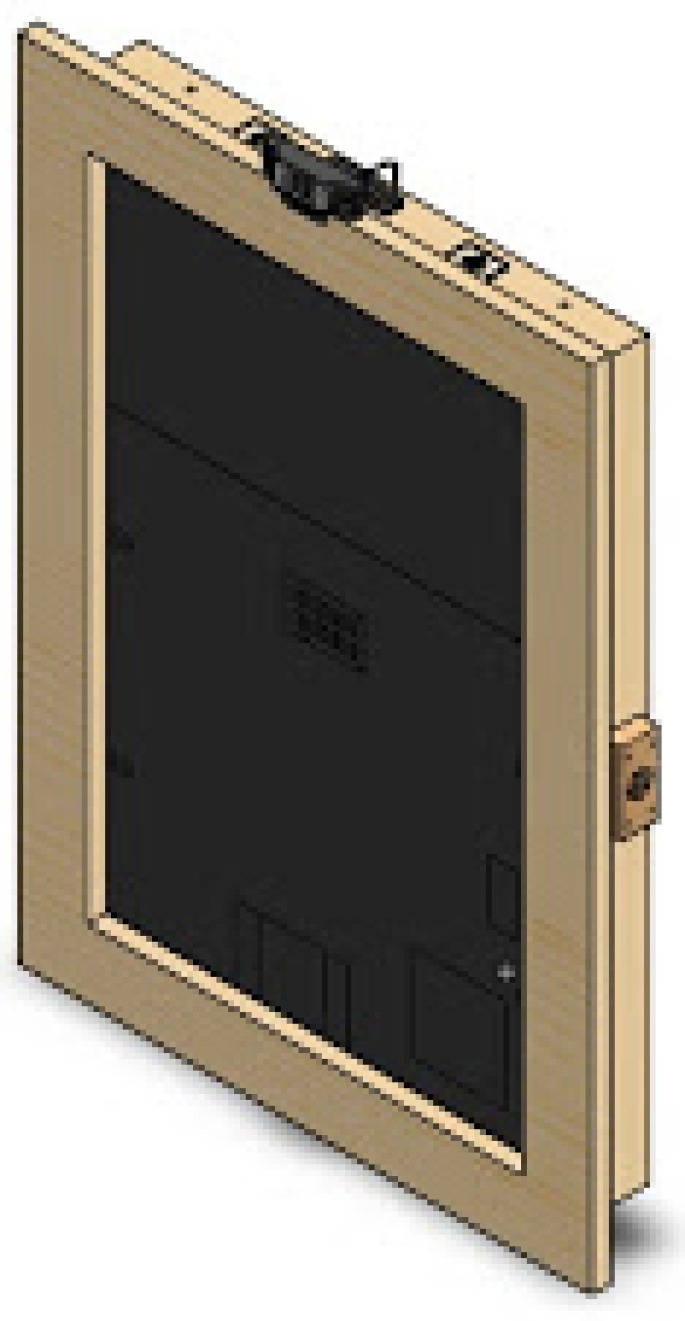
Smart mirror SolidWorks prototype.

**Figure 4:**
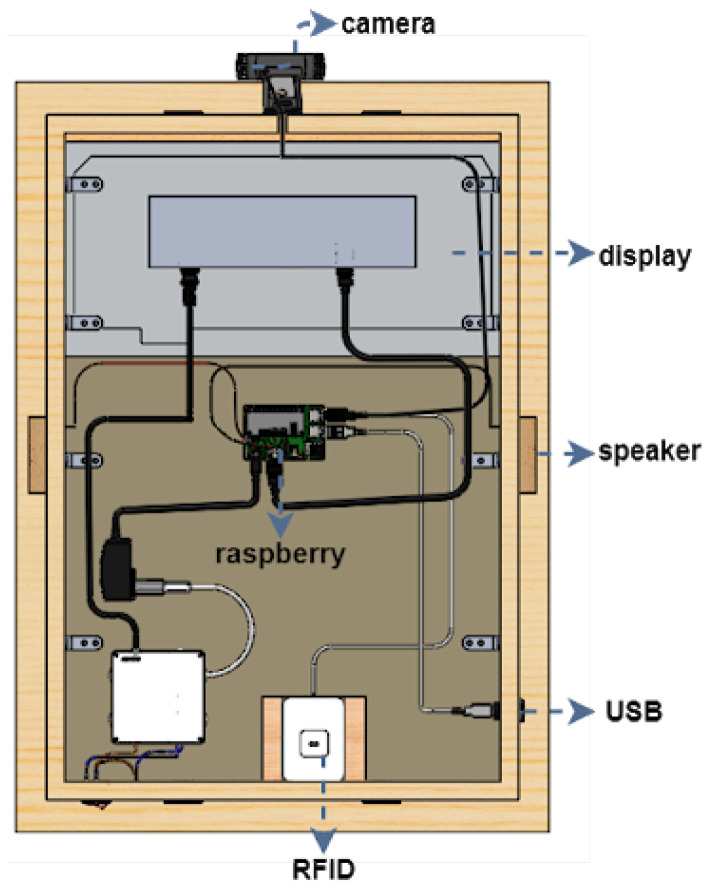
Component positioning at the back of the smart mirror platform.

The first version of the SHAPES smart mirror platform was designed fit-for-purpose. Different setups and materials were tested before reaching to the appropriate dimensions and materials that provides proper reflection (to give the mirror impression) as well as brightness so that under normal light conditions the information displayed in the screen could be easily visualized. This section summarizes the details of the first prototype, employed for the different tests described in this paper. The design is shown in the SolidWork (https://www.solidworks.com/ [accessed on 20 June 2021]) prototype depicted in Figure 3 and Figure 4. This design integrates the following components: a 22 inch screen, a Raspberry Pi 4, a Logitech C925 webcam, 2 W speakers, an RFID module and a USB input plug.

The Raspberry Pi 4 works as the computing node and is equipped with 8 GiB of RAM memory. This computing node supports the execution of the different digital solutions provided for the two pilot themes under evaluation. There are some storage functionalities that might also be delegated to private clouds. Specific Debian packages have been compiled and made available for an easy deployment. Furthermore, the automatic provisioning with Ansible has been implemented. These software implementations will be made available at the end of the SHAPES project.

### 3.3. The Smart Mirror Services

The SHAPES smart mirror provides a unique platform in which different applications can be deployed and run offering valuable services to promote healthy and active ageing. The major goals of the SHAPES smart mirror platform presented here are to support independent living and to facilitate physical rehabilitation at home, these objectives will be partially pursued by addressing three of the major risk factors determining older adults’ health and well being as known: social isolation, falls and physical inactivity. The following subsections describe the different software solutions devised to address such challenges. Additional information of these software solutions for implementation purposes and formatted as short recipes with code examples is available in the project documentation page (https://arcogroup.bitbucket.io/shapes/ [accessed on 25 November 2021]).

#### 3.3.1. The Call Service

The COVID-19 outbreak has caused a major and unprecedented health crisis. Efforts have been addressed to provide urgent medical attention to those going through the disease. Population confinement has been one of the most effective means to avoid the spread of the disease. This has, however, contributed to worsen the isolation situation already suffered by many older adults that live alone. This part of the population are, mostly technological illiterate, meaning that both access and use of technology cannot be provided without assistance. Communication technologies running on tablets, smart phones or computers have been designed with the general public in mind, overlooking the needs of a specific part of the population, such as older adults. Only a small portion of the older adults effectively use such communication tools.

The call service described here is therefore intended to address social isolation, in general, but particularly that suffered under a pandemic context (A short video can be seen in the following link showing the use of the call service: https://youtu.be/cin620HIkSQ [accessed on 24 November 2021]). The SHAPES smart mirror platform provides a perfect platform for video-conference purposes since it can handle both video and audio. Efforts have been therefore addressed to provide a call service that puts the focus on ensuring the acceptance and satisfaction of the older adult population. Following a co-designed approach, different participants and experts from the SHAPES project were involved through the different iterations of the design, implementation, and testing phases. This has ensured the usability, user acceptance and satisfaction of the resulting technology. The result is a video-call service built upon the Telegram network (https://telegram.org/z/ [accesed on 7 May 2021]). This service offers an interface specifically designed to run on the smart-mirror platform in such a way that minimum interaction is required to start a call. Although all functionalities of Telegram would be available, the focus has been made in providing a video-call service.

**Figure 5:**
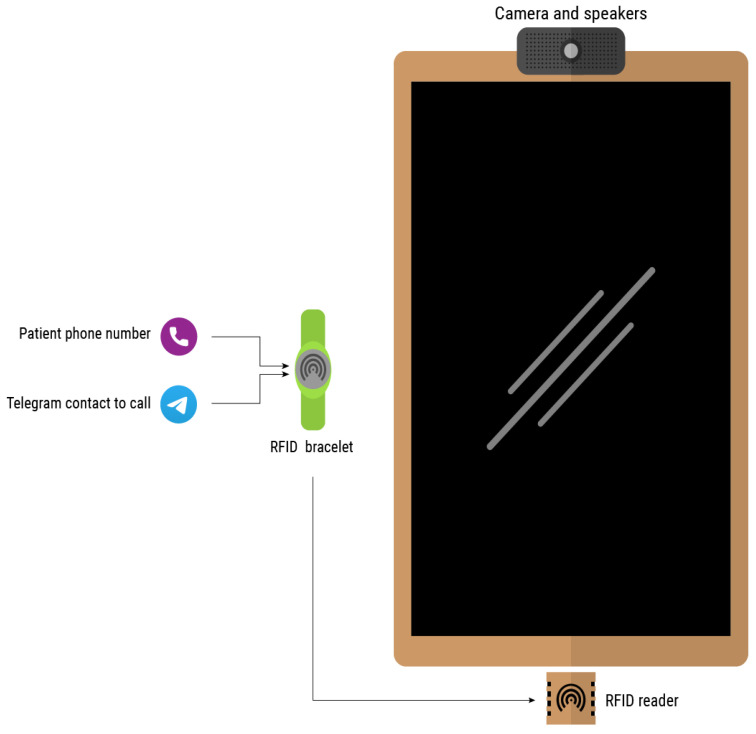
Elements intervening in the call service.

Users need their own telephone number, at least at the beginning of the configuration, as this will be the number associated when creating an account on the Telegram network. The contact(s) ones wishes to call also need to have an account on the same network. To simplify the call process, a system has been developed based on an RFID wristband which stores both personal information and contacts. The login or call process is initiated by approaching the wristband to the reader. If the system is not logged in, e.g., in scenarios in which the smart mirror is shared among multiple users, the first step would be the login process. Otherwise, it would directly read the contacts or, if it is only one, it would start the video call. Figure 5 depicts the elements intervening in the video call.

**Figure 6:**
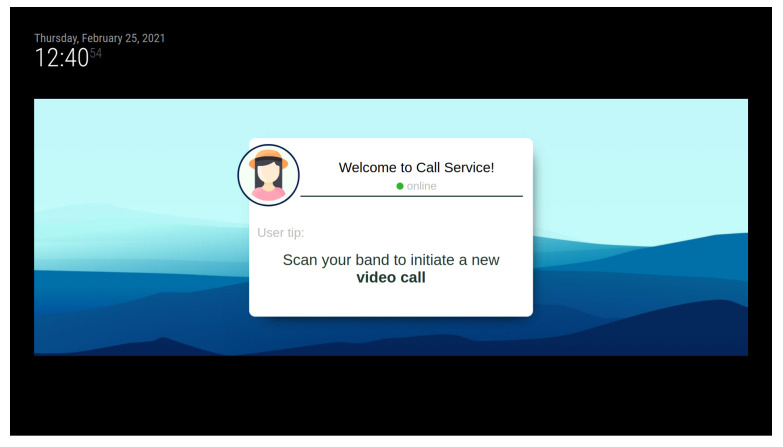
The call service interface.

**Figure 7:**
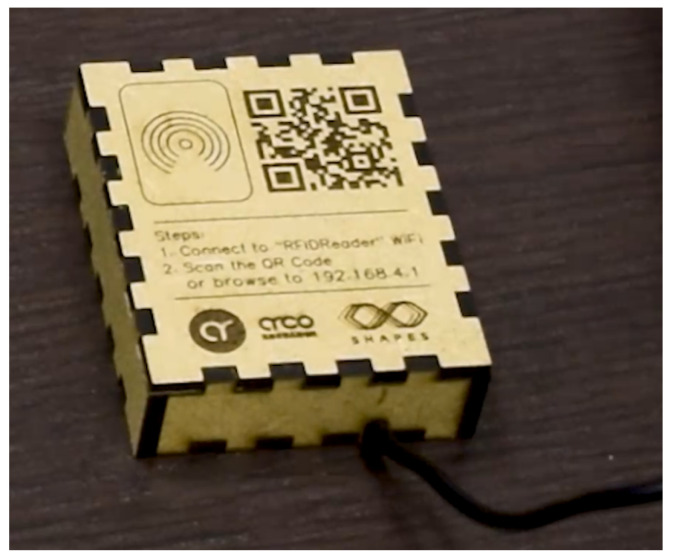
Wristband RFID writer device.

**Figure 8:**
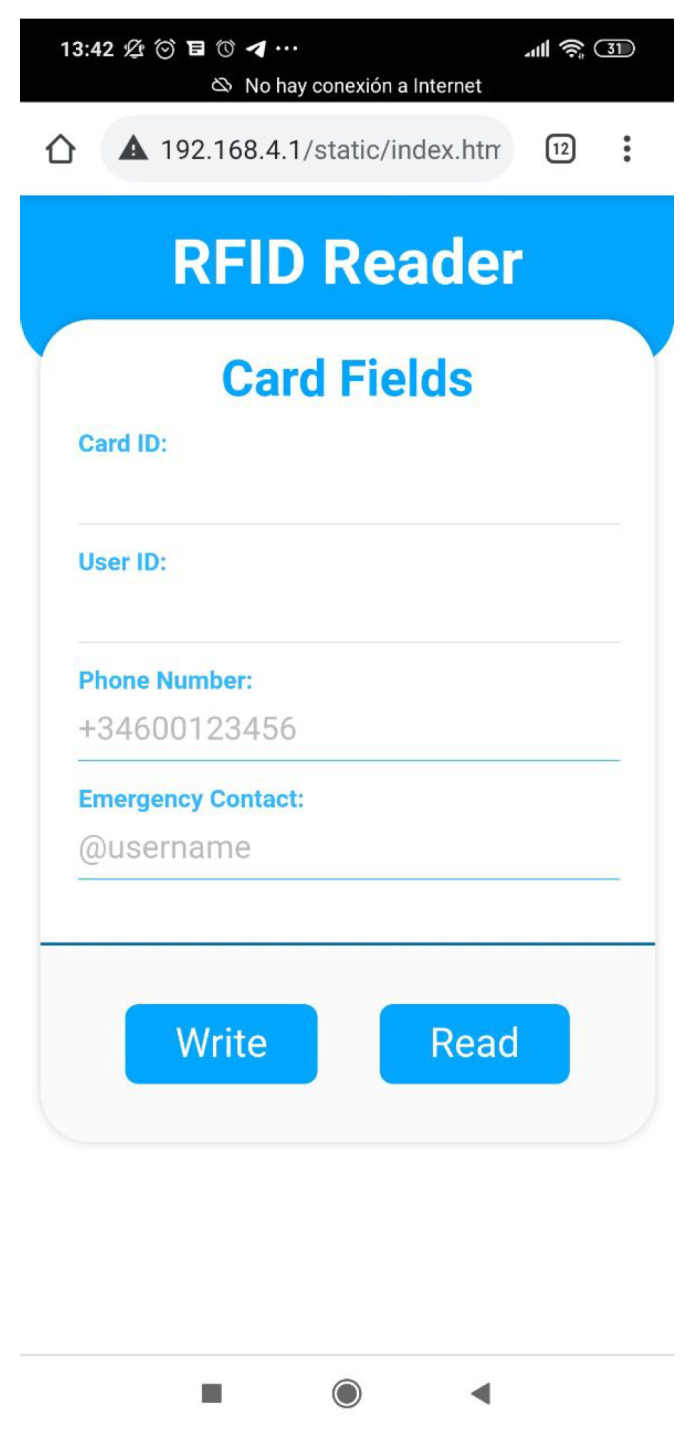
The interface for the RFID writer device.

This is an intuitive systems that requires very little interaction from the user, other than approaching the band and, in some cases, select the contact they want to call. Figure 6 shows the interface displayed in the smart mirror, requesting the band to be scanned in order for the call to start. Additionally, the process of burning information in the smart band is also very simple and intuitive. The device shown in Figure 7 has been designed to simplify the process. The QR code should be scanned with a smartphone. This opens a very simple interface, like the one depicted in Figure 8. When the smart band is placed in the labelled square and the information has been provided to the appropriate field, by pressing the *write* button the information will be written to the band.

#### 3.3.2. The Fall Detector

One of the major risks to be addressed, with the expected ageing of the population, are falls. They are one of the main causes of major injuries and even death in the most severe cases. To avoid or mitigate all these negative consequences, it is necessary to attend to the injured person as early as possible. The most vulnerable people are those who live alone. Technological solutions have already been created, such as push buttons that allow the user to notify others of a fall. These types of solutions, although useful, may not be fully effective in cases in which the victim is disoriented or may even lose consciousness. To meet this need, the smart mirror solution proposed here, provides an automatic fall detector system that, integrated with the call service, notifies the emergency contact when a possible fall is detected.

The fall detector operates as a service within the smart mirror platform. This service receives data from an external sensor connected via BLE (Bluetooth Low Energy). The sensor takes inertial readings from the person, thus being able to detect changes in movement such as those that would be caused by a fall. The sensor is placed at the user’s waist, as this is the centre of gravity of the human body. By placing the sensor there, any movement that is likely to cause a loss of balance leading to a fall can be easily detected. The sensing device used here is the MetaMotionR sensor from the manufacturer Mbientlab (https://mbientlab.com/ [accessed on 17 May 2021]), equipped with the following sensors: 3-axis Accelerometer and 3-axis Gyroscope. Besides these magnitudes, the sensor provides, by means of an internal algorithm, the absolute orientation of the sensor. This information can be very meaningful for fall detection, as it could indicate whether a user is on the ground. This absolute orientation is also found in all three axes of the sensor, and Euler angles are used as a measure.

The sensor collects data from the user’s movements and sends it back to smart mirror platform. The smart mirror collects this data, and from it, it will be able to recognise whether the user has suffered a fall. This process is based on a machine learning model, which has been previously trained with data from 17 different subjects. An SVM (Support Vector Machines) model has been used as it is the one that has the best performance in this type of problems. These 17 subjects performed different activities in a controlled environment, classified into ADLs (Activities of Daily Living) and falls (dataset publicly available (https://arcoresearch.com/2021/04/16/dataset-for-fall-detection/ [accessed on 13 October 2021])). From these activities, labelled data were generated and used to train the model to discriminate when a fall occurs. After testing the model, an accuracy of 100% can be reported in controlled environments.

**Figure 9:**
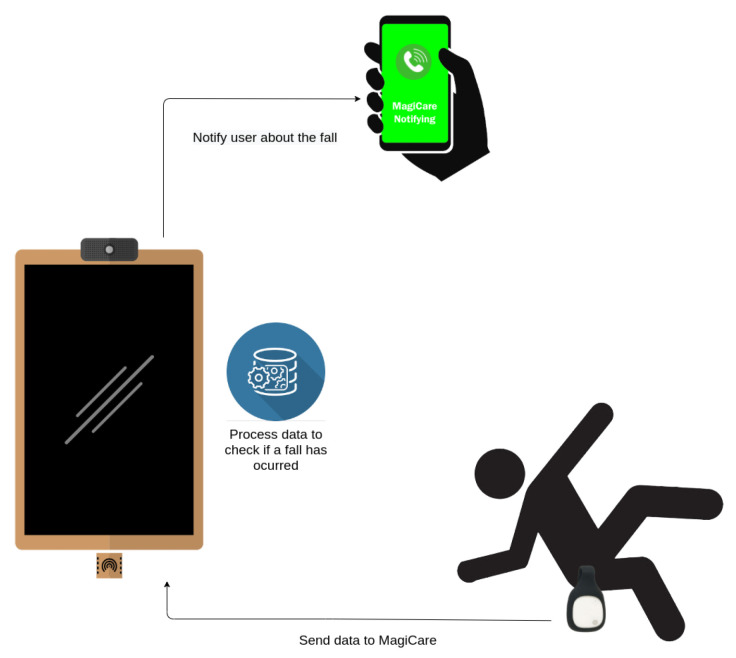
Fall detector.

The smart mirror, therefore, acts as a gateway, collecting data and determining whether a fall has been detected or not (Video illustrating the use of the fall detector: https://youtu.be/8Zn43OqBd1w [accessed on 15 October 2021]). If a failure is detected, the smart mirror notifies the user’s emergency contact via the call service (see Section 3.3.1). This enables prompt response to the fall, even in cases of disorientation or loss of consciousness of the victim. As this solution uses BLE, it is intended to monitor a person in the same environment in which the mirror is placed, as BLE has a limited range of approximately 5 m. The coverage range of BLE technology depends on the type of device. In general, devices used to monitor users in real time use class 2 devices, whose range is between 5 and 10 m. If a class 1 BLE device were used (which do have a range of 100 m) the sensor consumption would increase from 2.5 mW to 100 mW, so in these types of devices, in which energy efficiency is very important, class 2 is usually used. It is also possible to use coverage extenders if necessary. The system’s operation is shown in Figure 9.

#### 3.3.3. The Physical Activity Monitor

According to the WHO [47] “*at least 80% of all heart disease, stroke and diabetes and 40% of cancer could be prevented*” by tackling the most common risk factors underlying the most prevalent chronic conditions, such as unhealthy diets, physical inactivity, hypertension or obesity. Off-the-shelf devices and Apps can be found for physical activity and weight management such as those of Fitbit (https://www.fitbit.com [accessed on 12 June 2021]), Apple (https://www.apple.com/es/watch/ [accessed on 20 June 2021]), Google Fit (https://www.google.com/fit/ [accessed on 20 June 2021]) or Xiaomi Mi Band (https://www.mi.com/es/mi-smart-band-5/ [accessed on 20 June 2021]). They all offer a range of functionalities for user engagement, monitoring, reminders for promoting a healthier lifestyle, etc. Most of these commercial solutions offer open APIs, so that third party applications can access the data they collect. Thus, efforts can be focused on what to do with the data rather than how to collect them. However, a recent study [48] concluded that there is little evidence that wearable devices could improve health outcomes in older adults, although they could improve motivation and physical activity. Current approaches rely on wearable devices as enablers of behavioural change and most studies to date focus on healthy individuals rather than on those already suffering from a chronic condition or multi-morbidity.

The solution proposed here, named Phyx.io, take this technology a step further, enhancing the facilitating capability of *smartbands* with the potential functionalities that can be supported by the smart mirror platform. This combination enables more efficient interventions towards physical activities, which will eventually lead to better a health and well being.

Phyx.io is a platform which automatically manages and monitors many health-related parameters affecting older adults. This is carried out through the commercial Mi Band 4 smart band, providing information about the most relevant health parameters:

- **Activities:** The activities that the user has carried out in a 24-h period, among the following ones: asleep state, offline state, walking state and resting state.
- **Calories:** Calories burnt, based on the activity and intensity of the carried out activities.
- **Number of steps:** The number of steps taken by the user, automatically tracked.
- **Heart beat:** The system will automatically and repeatedly record the heart rate indicating the maximum value, the minimum value and the current value.
- **Sleep quality:** It considers two sleep states, namely: light sleep and deep sleep.

**Figure 10:**
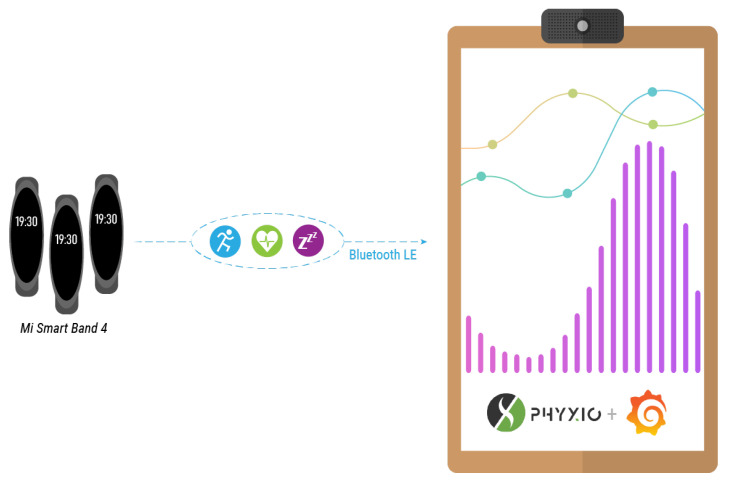
Physical activity monitor.

The Mi Band 4 smart band records these health parameters and stores them internally. This information is not sent to the Xiaomi cloud as privacy and data protection are major concerns of the smart mirror platform. The information is instead retrieved through a Bluetooth connection between the Mi Band 4 and the smart mirror, as depicted in Figure 10.

**Figure 11:**
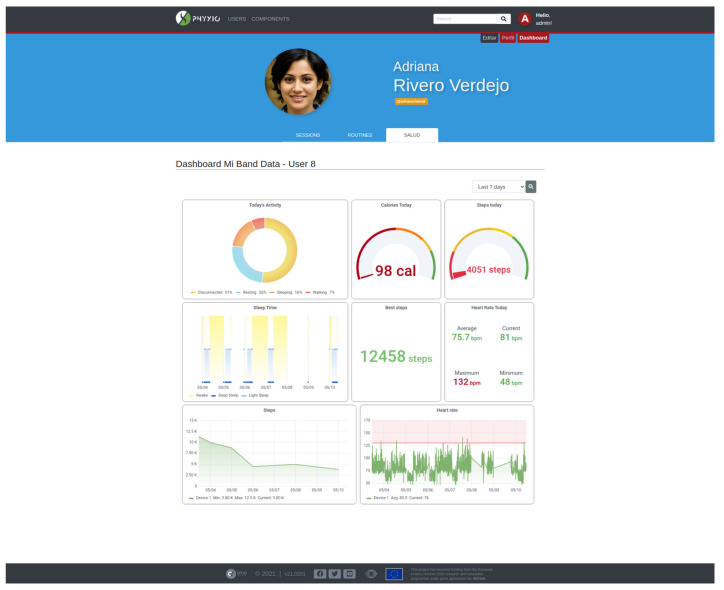
Dashboard of the physical activity monitor.

A service has been developed to establish a point-to-point connection between the Mi Band 4 smart band and the smart mirror. This service has been built on the Python library Pygatlib (https://github.com/oscaracena/pygattlib [accessed on 25 November 2021]). Once the information is retrieved from the smart band, it does not necessarily means that this has to remain locally in the smart mirror. It can be sent to a private cloud, from where the Phyx.io system can access it for displaying purposes. Phyx.io has a built-in dashboard, in which such health parameters can be explored, as shown in Figure 11.

Phyx.io does not only monitor physical activity parameters, but it also intervenes by making recommendations and by supervising the performance of physical-exercise routines intended to recover or maintain the physical condition.

Recommendations are made through the Mi Band 4 smart band. These can be intended to promote physical activity, either by encouraging to walk and achieve a step goal or by means of remainders of a scheduled physical exercise routine. Recommendations can also be addressed to ensure the general well being, such as to drink water recommendations on hot days or general remainders. These recommendations are configured by the caregiver or the therapist.

**Figure 12:**
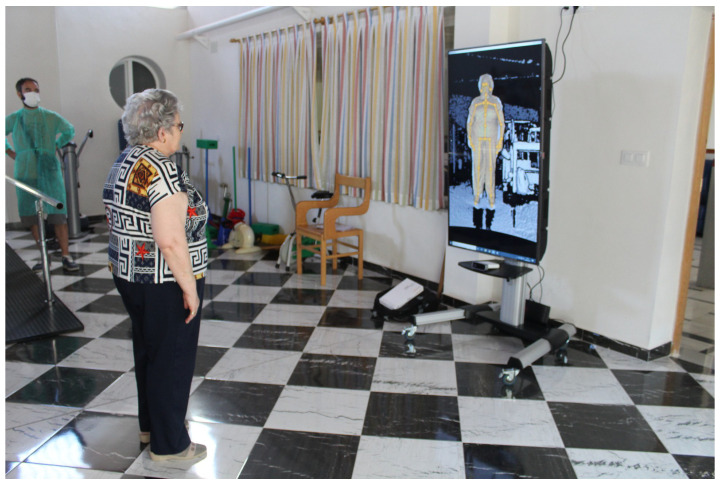
Phyx.io physical rehabilitation kiosk.

Phyx.io supervises the performance of physical exercise routines prescribed by a therapist. The purpose of these exercise session can either be the recovery from an injury or just to stay physically active. The recovery routines are categorised into orofacial rehabilitation exercises and the exercises with the rest of the body. For the latest, a kiosk version as the one depicted in Figure 12 has been devised. Equipped with a depth camera, Phyx.io supervises and corrects the performance of the different exercises comprising a routine. The system is an advanced version of the one described in [49] (Video of the ArthriKin rehabilitation tool: https://youtu.be/tlDppOljiq0 [accessed on 25 November 2021]) since it also incorporates a new functionality for orofacial rehabilitation.

For the orofacial rehabilitation exercises a lightweight computer vision-based system has been developed, using three Deep Neural Networks (DNNs) to detect faces, facial keypoints and the considered facial gestures, respectively. The deployment of these DNNs is optimized for mobile/IoT platforms using MediaPipe and TensorFlow Lite for the inference. First, the user’s face detection is performed by BlazeFace [50]. This DNN is related in structure to MobileNetV1/V2 [51,52] and the Single Shot Multibox Detector (SSD) framework [53], and is aimed at effective GPU utilization in mobile/IoT devices. Its output is used to initialize another DNN [54], which allows inferring a dense 3D mesh representation of the user’s face. Thus, based on the localized facial keypoints we rotate and rescale the facial images into normalised face patches in order to minimize the impact of facial image shape and appearance differences that could affect the subsequent gesture recognition stage.

**Figure 13:**
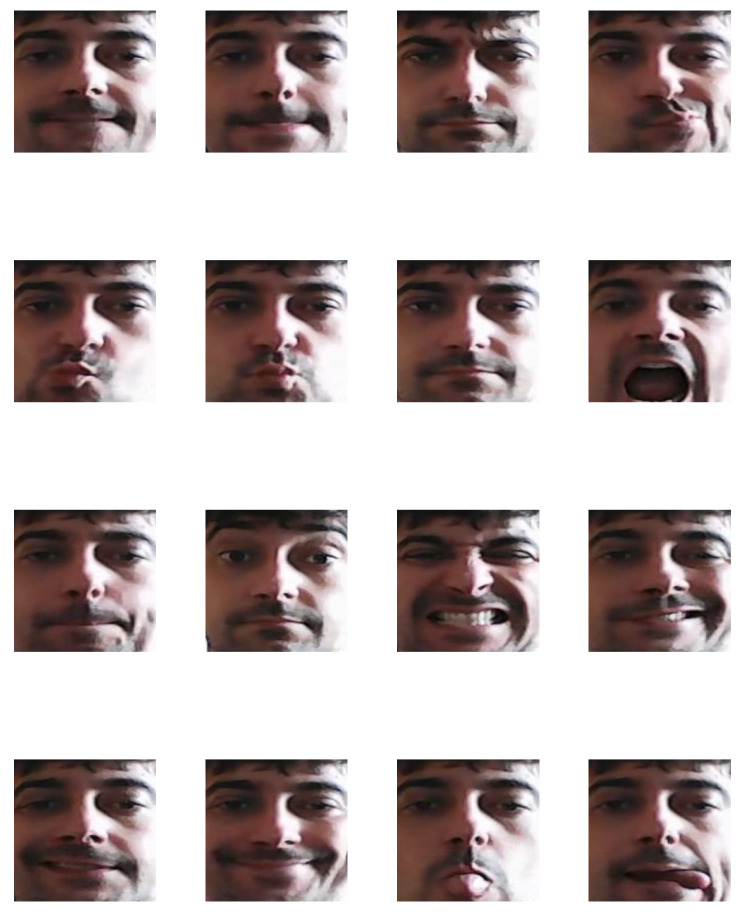
Examples of reference orofacial gesture images.

In this setup we have considered the following 17 gestures for orofacial rehabilitation: *bite_lower_lip*, *bite_upper_lip*, *frown*, *kiss_left*, *kiss_right*, *kiss*, *neutral*, *open_mouth*, *press_lips*, *rise_eyebrows*, *show_teeth*, *smile_left*, *smile_right*, *smile*, *tongue_forward*, *tongue_left* and *tongue_right*. We have extracted normalised face patches from 19 people performing several repetitions of these gestures in uncontrolled ’in-the-wild’ environments to train a DNN for image classification based on EfficientNet-lite0 [55]. This DNN is the most efficient version of the EfficientNet-lite mobile/IoT friendly image classification models. Its output is a 17-dimension vector of floating-point numbers that go from 0 to 1. This trained model serves as the reference guide for users. Thus, if the user performs one gesture close to the learned reference, the output will be close to 1 for that gesture. This way the system can automatically evaluate how well the user is performing every gesture. Thresholds could be configured to determine whether a user performs sufficiently well the gestures in real time and to analyse the evolution through time. Figure 13 shows some examples of the recorded reference images.

The physical therapist or trainer plays a key role, not only prescribing exercise routines but also supervising the evolution, especially under rehabilitation processes. To this end, Phyx.io offers a dashboard view with records over time. The idea is that, even if the Phyx.io system corrects wrong body posture or assists the user during the routine, experts can latter on access the information generated during the routine. This can help evaluating adherence to the prescribed routine, level of achievement (for example, based on the number of repetitions prescribed and actually performed), accuracy of the performed exercise measured against the baseline pattern, time, etc.

Finally, it has been demonstrated that older adults receive less social support and have more fear of falling during physical activity [56]. This can lead to a poor adherence to exercise programs to users living alone, both for the lack of social support, motivation or the fear of a fall going unnoticed.

To address the lack of social support, Phyx.io is enhanced with capabilities for a one-to-one communication with physical therapist, trainer or an exercise partner. The idea is that users perceive exercising with Phyx.io almost as if they were training in a gym with the expert or other users by their side. First sessions are prone to demand expert support, or the need to feel accompanied, specially to address doubts. The call service has been embedded in the Phyx.io interface in such a way that video calls can be initiated during the performance of an exercise, or in a scheduled manner. This is very useful not only for user adherence but also for experts to receive feedback from users.

The fear of falling is addressed here by the fall detection system presented in Section 3.3.2. Users wear the MetaMotionR sensor in their waist so that when a fall is detected by the system, a call is launched through the call service to the emergency contact.

#### 3.3.4. The Voice Assistant

The conversational interface provided by voice-based digital assistants is more intuitive and easier to use than hand-keypad or touch based input interfaces [57]. A voice assistant, embedded in the SHAPES smart mirror, could therefore provide a natural-language based interaction. Nevertheless, privacy and security concerns have been recently arisen with regard to the use of this technology by vulnerable users, such older adults [58].

Digital assistants such as Siri, Alexa and Google Assistant based their strengths in the currently available processing power and the large amount of information these companies have been able to collect and process to train advanced machine learning models that deliver personalised services [59]. Nevertheless, the use of such technologies comes with a *privacy trade-off*, similar to the one stated for the health recommender systems [60]. A work-around to this privacy trade-off is to provide our own voice assistant, similarly to the approach we have previously describe for the home and physical activity monitoring systems.

**Figure 14:**
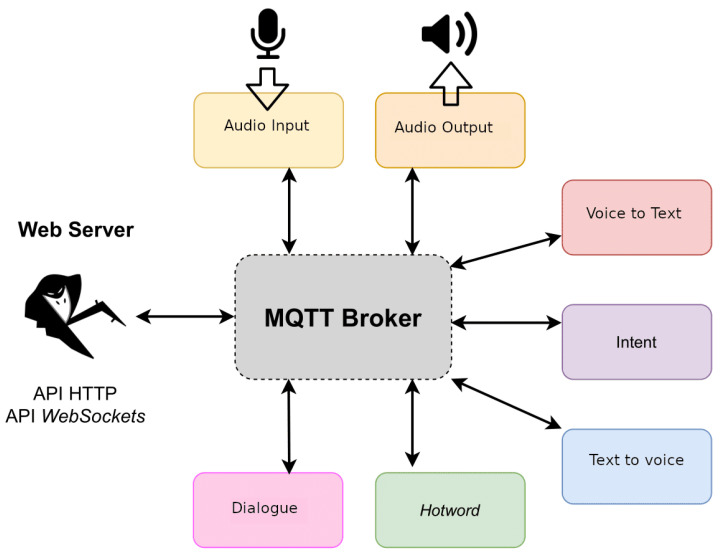
Services provided by Rhasspy for the voice assistant.

The proposed architecture is based on the use of a set of voice assistant services provided by the Rhasspy tool (https://rhasspy.readthedocs.io/en/latest/ [accessed on 18 May 2021]). From all the services provided by Rhasspy (see Figure 14) the proposed solution just employs the speech to text transcription service, the intent recognition service and the intent handling service. For speech recognition, a model for the Spanish language has been trained with DeepSpeech which is a speech recognition engine based on a trained model using machine learning techniques (https://github.com/mozilla/DeepSpeech [accessed on 18 May 2021]).

**Figure 15:**
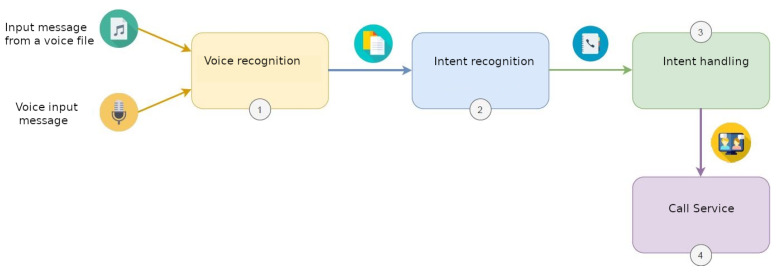
Voice assistant workflow.

The proposed solution follows the steps depicted in Figure 15, where the voice assistant provides an intuitive manner of accessing the video-call service, other than using the RFID wristband, by taking the following steps:

1. The audio input that is received is transcribed with the trained Spanish model and the speech recognition service. In this case, the audio input is the phrase quoted by the caller and the name of the contact with whom he/she wants to make the call.
2. The intention is recognised with the intention recognition service and mapped against the intention to make a call.
3. The call service is launched via the intention handling service, by triggering the command that perform a call to the contact stated in the audio input.
4. The call service starts the call.

#### 3.3.5. Calendar with Reminders

Multi-morbidity is highly prevalent, reaching up to 90% of people over age 65 [61]. This is, at the same time, very challenging because different professionals are involved in the treatment, medication is complex and involves polypharmacy with a high risk of drugs interfering with each other, or poor medication adherence. According to [62] polypharmacy is usually associated with poor adherence. Non-adherence, when unintentional, may be due to forgetting or lack of reminders [63]. The work in [64] analyses the different applications found in the Apple App Store and the Google Play Store. The use of medication reminders were positively perceived by users. In this sense, different approaches can be found in the literature such as the work in [65] that proposes a system based on SMS reminders or the one in [66], in which a calendar with reminders is presented.

The use of reminders is not only relevant for medication adherence purposes, as it has also been demonstrated its positive impacts in adherence to physical activity interventions [67]. More specifically, the work in [68] proposed an approach based on the self-efficacy theory [69,70]. This approach consists of stating realistic and attainable goals combined with incentives in the shape of motivators or reminders. Following this approach, the SHAPES smart mirror platform provides support for reminders that complement the reminders also provided through the Phyx.io service, sent to the Mi Band 4 smart band.

This service displays events from two different calendars, one at the organisation level and one at the person level. The first category covers events that affect all users in the context of a multi-user smart mirror (i.e., smart mirrors in a nursing home context). This might involve the event regarding an activity that takes place in the nursing home and to which all residents can attend. It also covers events that limit the use of the mirror, such as an appointment made for a video call, again in the context of a smart mirror with multiple users. It is important to note that all events registered in this calendar will be public, i.e., they will be displayed in the smart mirror interface without previous authentication.

The second calendar handles events concerning a single subject, like those regarding medication reminders or relatives’s birthdays, for example. This calendar therefore requires the person to be authenticated with the RFID wristband mentioned during the description of the call service in Section 3.3.1.

**Figure 16:**
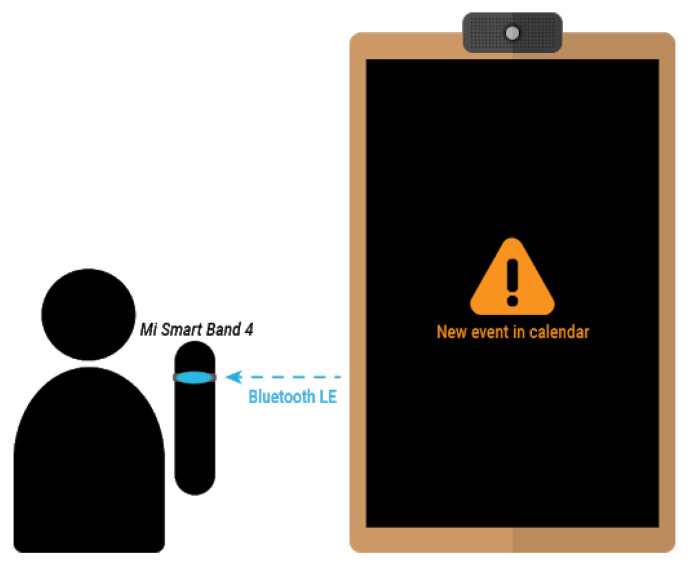
Reminders sent to the Mi Band 4 smart band.

Apart from using the smart mirror interface, notifications can also be sent to the Mi Band 4 smart band, as described in Section 3.3.3. This notifications will cause the smart band vibration, along with a description of the event and the scheduled time. In order to receive such notifications, the user has to be within the range of the smart mirror or any of its replicas working as a signal extender, as depicted in Figure 16.

#### 3.3.6. The Home Monitoring System

The SHAPES smart mirror platform has been originally devised to support and extend independent living for older adults suffering neurodegenerative as well as to support at-home physical activity and rehabilitation routines. To this end, the knowledge obtained from monitoring the home environment and the behaviour of the older adults that live there is essential.

Human action recognition has been, for a long time, one of the major challenges faced by scientific community. Different strategies have been employed, affecting privacy in different ways, such as those based on video, audio and/or sensors. Video-based and deep learning techniques have experienced relevant advances in assisted living [71], mainly due to the richer information provided by cameras [72]. However, privacy expectations are high [71] in the home environment and although safety is preferred over privacy, the context where the camera is located does impact on the technology acceptance [73].

The concept of privacy is broad [74], as it encompasses different dimensions, such as physical privacy, psychological privacy, social privacy and informational privacy. A good balance between functionality and privacy concerns, as well as cost, is essential for a successful adoption of technological solutions [17]. Although cameras provide richer information about the context, such level of detail is not always needed. In such situations, the trade-off between privacy and functionality favours privacy over the second. This is the case here, and for this reason an approach solely based on sensors is proposed for the home monitoring system. However, the use of sensors also comes with a price in terms of privacy as device interoperability has traditionally been addressed by using manufacturer’s private cloud. It is therefore necessary to devise a mechanism to ensure device interoperability, specially among different vendors, avoiding the use of vendors’ cloud. To this end, an architecture is proposed that has the SHAPES smart mirror at its core, to perform data collecting, processing and storage.

**Figure 17:**
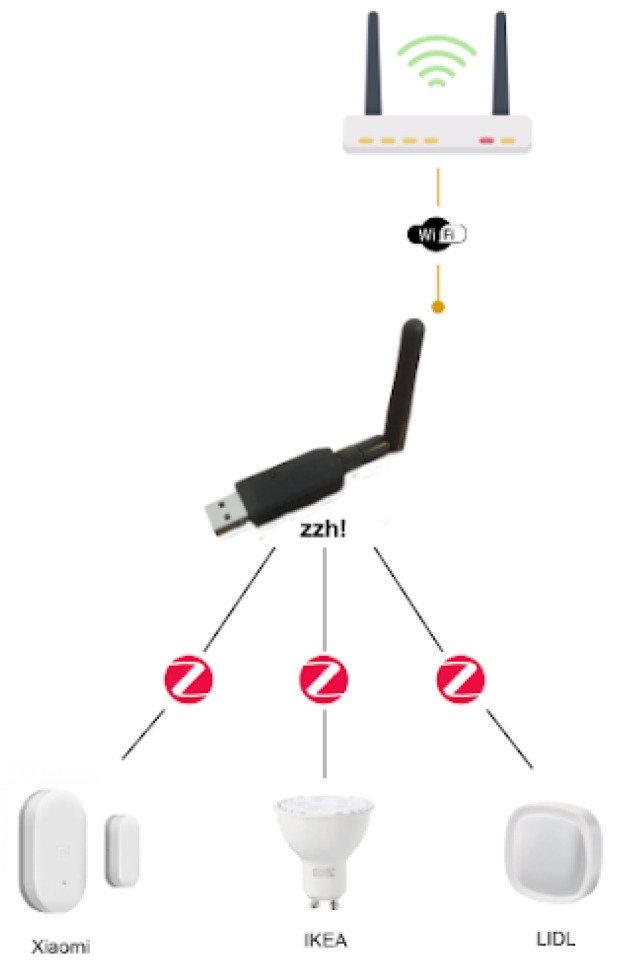
ZigBee devices coordinated by a zzh!

The use of different communication technologies is also an aspect to be considered as this has an impact mainly on interoperability and price. Table 7 compares the most common technologies used for smart home monitoring. Among them, mainly because of the sensor variety and cost, the ZigBee technology stands out as the most appropriate one for the purpose of supporting independent living for older adults with neurodegenerative diseases. Moreover, it also guarantees a low cost and low energy consumption, with the possibility of using sensors from different manufacturers. Nonetheless, despite using the same communication technology, different manufacturers using ZigBee would still require their own hub or gateway to support their devices. Luckily, an open-source project, known as ZigBee2MQTT (https://www.zigbee2mqtt.io/ [accessed on 23 June 2021]), provides a way to interconnect devices of different manufacturers with ZigBee connectivity. It allows ZigBee devices to be controlled via MQTT (https://mqtt.org/ [accessed on 23 June 2021]) through an event bridge. This requires a computing node to run the event bridge. This computing node needs a ZigBee interface. This type of interface is not very common, in fact, the Raspberry Pi 4 lacks of such communication interface. This limitation can be easily overcome thanks to devices such as the zig-ah-zig-ah! or zzh! device (https://www.zigbee2mqtt.io/information/supported_adapters.html [accessed on 23 June 2021]). This is a small development board in the form of a USB stick, compatible with ZigBeeMQTT, which provides the ZigBee communication interface to our computing node, turning it into a gateway, therefore working as a protocol coordinator. The use of both elements results in a much more cost-effective option for connecting a wide range of devices. Figure 17 summarizes the proposed architecture.

Most of the state-of-the-art platforms for IoT are cloud-based solutions. The use of the cloud offers a simple way (although not the most efficient one) of addressing the heterogeneity (in terms of devices and networks) in IoT. Many inconveniences are, nevertheless, derived from implementing entirely-cloud-based solutions such as high latency, resource consumption, low fault tolerance, or unnecessary data transportation for services that support their responses on local information, without needing aggregated or historic data. Another major inconvenience of a cloud-based solution arises when the cloud is owned by the manufacturer, and control is lost over its processing. The solution proposed here overcomes these limitations by using the SHAPES smart mirror platform as a data hub where, after a pre-processing stage, decisions are made about locally storing or pushing the data to a private-cloud solution, after going through the appropriate mechanisms to ensure data privacy and security issues.

**Figure 18:**
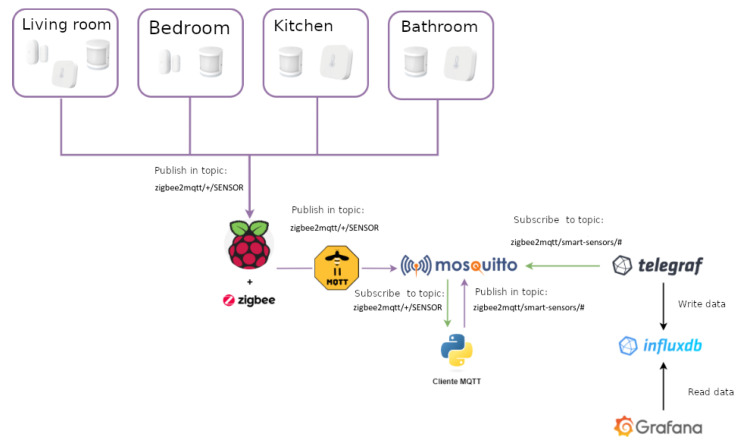
Architecture of the home monitoring system.

The implemented system is based on the use of a number of sensors, including: motion sensors, door and window sensors, and temperature and humidity sensors, as depicted in in Figure 18.

The proposed architecture has been implemented using the ZigBee wireless communication protocol and the MQTT messaging protocol for transporting the data collected and published by the sensors. A Telegraf client (https://docs.influxdata.com/telegraf/v1.19/ [accessed on 20 May 2021]) has been used as a subscriber to the topics created for the different rooms of the house where sensors are located. The data is stored in a temporal database, either locally or in the cloud, using for this purpose the InfluxDB database (https://www.influxdata.com/ [accessed on 20 May 2021]). The integration of InfluxDB and Grafana (https://grafana.com/ [accessed on 20 May 2021]) is direct, so a visualisation solution is obtained in a simple and efficient way. The dashboard offers information that is easy to interpret for both the older adults and their caregivers.

The SHAPES smart mirror works as the hub or a gateway. Although initially, the smart mirror lacks of ZigBee communication support, the use of the zzh! coordinator enhances it with a ZigBee interface. The ZigBee2MQTT system listens the values measured by the different sensors and publishes them in the corresponding topic. The publication consists of a message with the event structure provided by MQTT, organized by entities and attributes. In this case, the entities are the sensors used, while the attributes are the data emitted by the sensors themselves.

The use of Telegraf is proposed to collect, process and send the data metrics provided by the different sensors to a database. In the architecture proposed here, the Telegraf agent subscribes to the topic “zigbee2mqtt/smart-sensors/#”, where the data collected by the sensors is published. InfluxDB has the structure of the event channel attributes. Finally, the Grafana data analysis platform is connected to the InfluxDB database to enable the visualization of the stored data. Grafana is configured to provide a dashboard with as many panels as rooms in the monitored home (living room, kitchen, bedroom and bathroom, for example). Each of these panels shows the collected values.

## 4. Results and Discussion

The SHAPES project is undertaking a pan-European pilot campaign plan. This plan follows a human-centred co-creating and co-design approach meaning users and relevant stakeholders have been involved all over the different phases of development or throughout the entire technology life cycle. User needs have been captured following this approach through the development of personas, user stories and use cases.

The SHAPES project has considered seven pilot themes and different pilot sites where such themes will be evaluated. Among these pilot themes this work has focused on Pilot Theme 5 “*Caring for Older Individuals with Neurodegenerative Diseases*”, which aims to improve the delivery of care, the management of health conditions and activities of daily living, and the communication and social well-being of individuals with mild cognitive impairment. More specifically, the use case considered is focused on providing a digital assistant for older people with mild cognitive impairment. Additionally, Pilot Theme 6 “*Physical Rehabilitation at Home*” has also been used in this work, which is intended to provide the appropriate tools to facilitate physical rehabilitation at home. This pilot theme will therefore address older individuals that need to recover from a certain accident or health issue (e.g., stroke, fall, surgery, arthritis rheumatoid, osteoarthritis, etc.), but also those who, without having to recover from a specific problem, benefit from physical activity. Pilot Theme 6 considers four use cases, although this work focuses on the three uses cases supported on the smart mirror platform, namely:

- Use case 2, training of orofacial musculature.
- Use case 3, video-based rehabilitation tool.
- Use case 4, wearable motion monitoring devices.

Different criteria (key performance indicators) have been established to evaluate the success of the different interventions devised for the considered pilot themes and use cases. The following criteria will be used:

- Recruitment and retention:
  - At least 80% of the target cohort (older adults) were successfully recruited into the pilot during the recruitment period.
  - At least 80% of recruited participants within the target cohort remained enrolled in the pilot until the end of the study.
- Technical performance:
  - No functional bug is reported by users in the last phase of the use-case pilot deployment.
  - A total of 100% of functional bugs are fixed in the pre-trials phase.
  - The overall technical solution assures 90% of availability.
- User engagement and acceptance:
  - The overall user experience of the Phyx.io tool using the short version of the User Experience Questionnaire (UEQ-S) was classified as ‘Excellent’, ‘Good’ or ‘Above average’ based on published benchmark data.
  - At least 80% of the older people under a rehabilitation routine program stick to it.
  - At least one care provider/caregiver scored the Phyx.io functionalities above average rating (>68) in the System Usability Scale (SUS).
- Other indicators (examples, to be defined in a measurable manner):
  - Older people: type of interaction with Phyx.io and duration.
  - Caregiver/Health Care Professional: number of routines assigned/reviewed each day.
  - Caregiver/Health Care Professional: number of videocalls scheduled per week.

This analysis is out of the scope of this article and will be part of the future work. Nonetheless, this work focuses on evaluating the performance and success of the different technologies designed to support such intervention.

An experimental validation approach is proposed based on the different use cases designed for the considered pilot themes. The following subsections describe each of the considered use cases along with the validation carried out. Moreover, because the smart mirror platform is based on a Raspberry Pi 4 device and its computational resources are limited, the first subsection starts by assessing the performance of such platform when running the different services.

### 4.1. The SHAPES Smart Mirror Platform Validation

Regarding the smart mirror platform, as an embedded system, its performance has been evaluated in terms of resource consumption and response time. The CPU parameters from Table 8 measures the percentage of the CPU time employed for the execution of the process, out of the total time during which the process was being analyzed. Moreover, the memory parameter from the same table is employed to measure the percentage of main memory employed by the process.

The smart mirror platform ecosystem is under development and it is expected to offer more services, so it is important to know what the computational load is with the currently implemented services and what is the margin it can have for the integration of future services. In order to know the load supported by the mirror, it was monitored for a period of 24 h to know if the use of resources is efficient or, on the contrary, the platform is being very strained. Throughout these 24 h, the assessment service performed measurements every 10 min, calculating the CPU usage in that interval and the memory usage.

The results after the monitoring time were the following. In terms of the overall system, an average CPU usage of 3.56% was calculated, indicating that proper use is made of processing resources, with the possibility of increasing the work with future services. Memory usage averaged 32.2%, which is slightly more negative than CPU usage, but not critical at the moment. The temperature of the processor was also measured, as high temperatures can indicate very demanding loads or even malfunctioning of a component. During the monitoring period the temperature remained around 50 °C, showing normal operation.

Additionally, besides monitoring the general system, individual processes were also monitored, so that in the event of finding any incidence in the overall system, it would be possible to study which processes were causing the performance problems. Among all the processes comprising the current SHAPES smart mirror solution, three stood out with the following data shown in Table 8.

None of these services were characterised by intensive use of resources, only the magic-mirror-2 and fall-detector services stand out in terms of memory usage, although this does not seem to be a relevant problem for the time being. The performance analysis confirms that the solution presented is quite efficient in the use of resources, offering the capacity to extend the services that comprise the SHAPES smart mirror platform.

### 4.2. Use Case 1: Digital Assistant for Older People with Mild Cognitive Impairment

**Figure 19:**
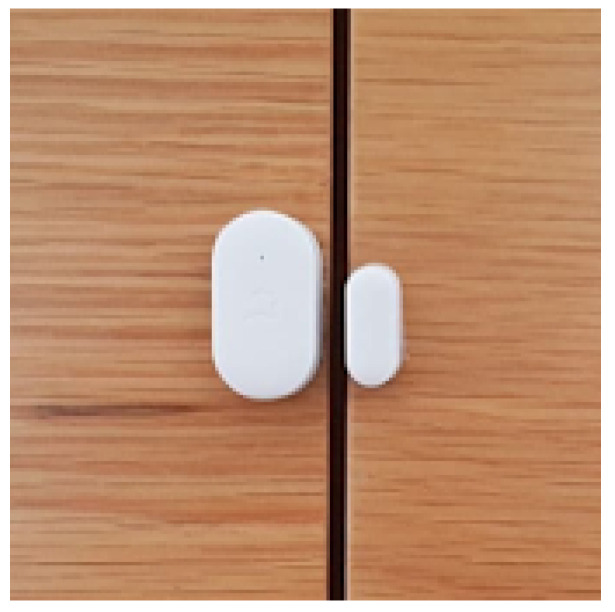
Door and window sensor to detect opening and closing events and the current state.

**Figure 20:**
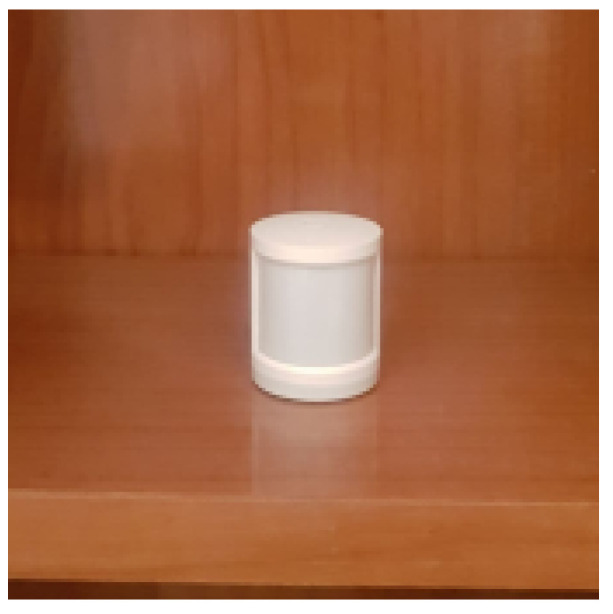
PIR-type movement sensor to detect presence in a room.

**Figure 21:**
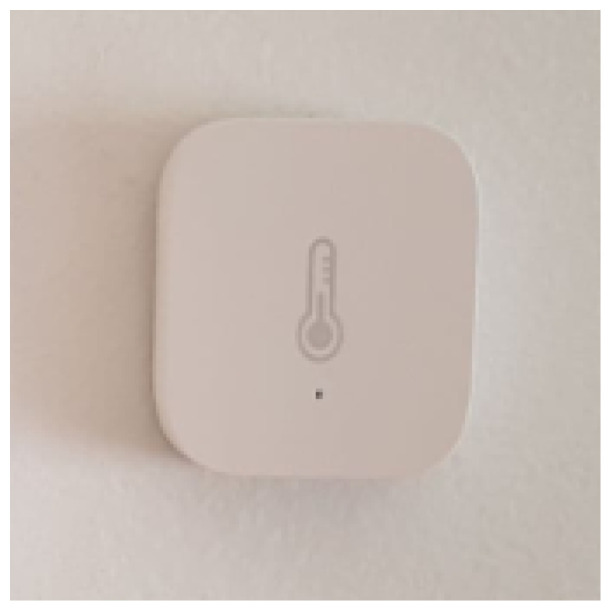
Temperature and humidity sensor to monitor comfort in a room.

**Figure 22:**
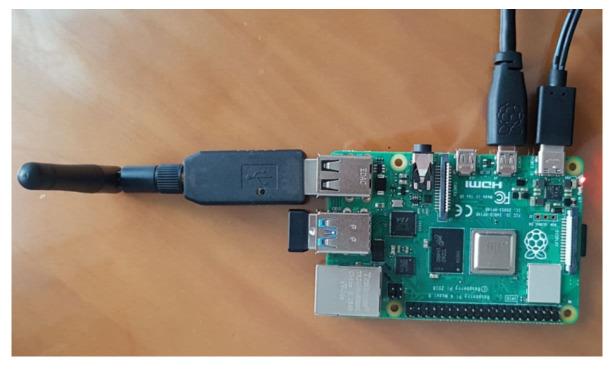
Raspberry pi 4 and zzh! providing gateway functionalities to integrate home sensors.

In order to validate the system performance under a realistic environment, a private home was recruited and equipped with following hardware devices:

- Xiaomi MiJia sensors for door and windows (see Figure 19).
- Xiaomi MiJia movement sensors, (see Figure 20).
- Xiaomi Aqara temperature and humidity sensors, (see Figure 21).
- Raspberry Pi 4.
- A Zig-ah-zig-ah! (zzh!) (see Figure 22).

**Figure 23:**
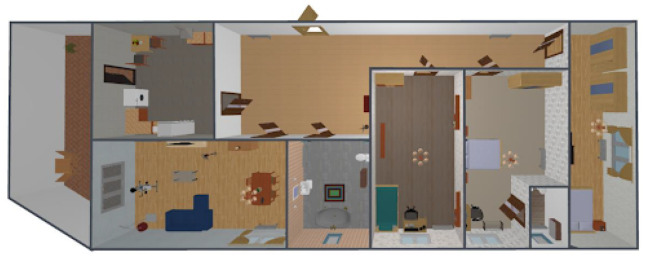
Floor plan of the home.

The selected sensors were deployed and configured to work in a private and real home, as the one depicted in Figure 23. This home has four rooms, namely: the living room, the bedroom, the kitchen and the toilet.

**Figure 24:**
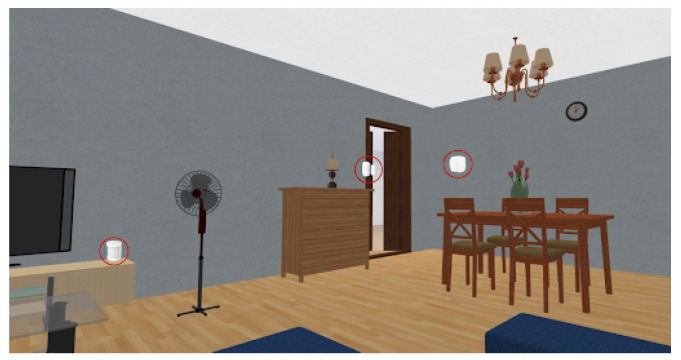
Sensors deployed in the living room.

**Figure 25:**
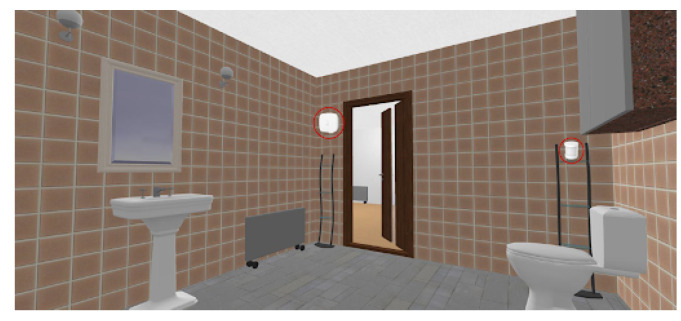
Sensors deployed in the toilet.

**Figure 26:**
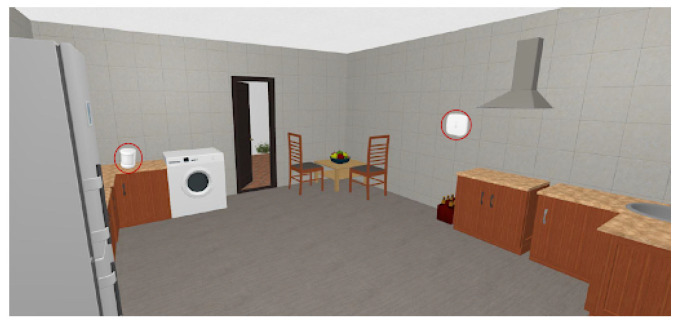
Sensors in the kitchen.

**Figure 27:**
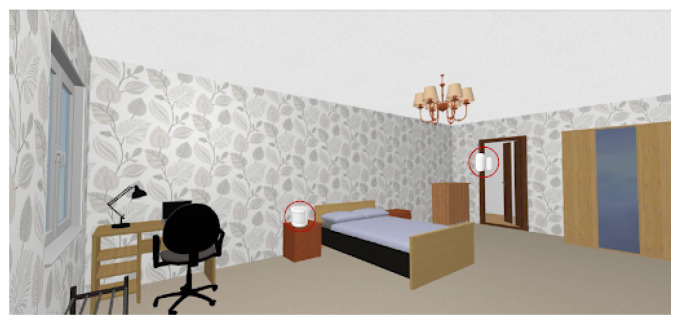
Sensors in the bedroom.

The living room was equipped with a movement sensor, a door and window sensor and the temperature and humidity sensor. The toilet was equipped with a movement sensor and a temperature and humidity sensor. The kitchen was equipped with a movement sensor and a temperature and humidity sensor. The bedroom was equipped with movement sensors and door and window sensors. The location of all those sensors is depicted in Figure 24, Figure 25, Figure 26 and Figure 27, repectively.

The smart mirror computing node, represented here by the Raspberry Pi 4, is equipped with the zzh! coordinator. This device enhances the smart mirror platform with ZigBee communication. This platform is installed in the living room covering the whole house.

The validation experiment has run for two months (March and April 2021), during which sensor events were collected. in total, 17,456 events were collected, corresponding to 11,560 from the presence sensor, 1231 of them from the door and windows sensors and 4665 events from the temperature and humidity sensors. From the analysis of the information collected during this experiment, the following conclusions have been drawn:

- The temperature of the dwelling has not suffered any important variation, always in the range of 20 °C and 26 °C and humidity between the 47 %H and 52 %H.
- The movement sensors provides information about where, in the dwelling, is the person located and therefore, carrying out activities. Between 9:00 a.m. and 2:00 p.m., no movement is detected in the dwelling. The person leaves the house for work. Then, between 23:00 p.m. and 7 a.m. only the bedroom area detects movement. The person is in the bedroom either sleeping (when no movement is detected) or preparing for it. During the afternoon, the main activity is located in the living room, apart from the periods during which the person visits the kitchen for meal preparation and eating.
- Regarding the door and window sensors, the information they provide is that doors are, most of the day, open, apart from the cooking or sleep time.

**Figure 28:**
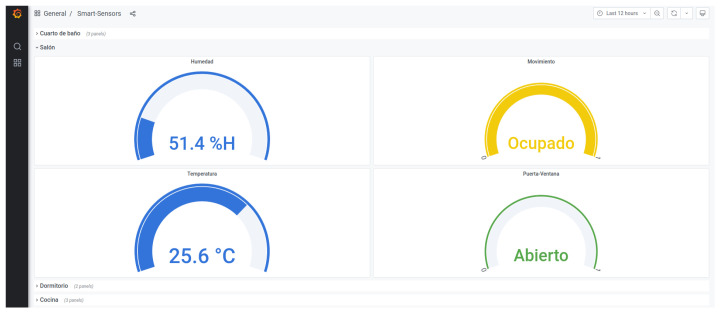
Dashboard for environment monitoring.

The dashboard depicted in Figure 28 shows an example of real-time remote monitoring. This interface is thought for the caregiver to know about the environmental conditions of the dwelling or the state of a certain door or window. Furthermore, historic records can be retrieved for further analysis intended to identify behavioural patterns that help an early recognition of behavioural changes consistent with cognitive decline.

Despite the simplicity of the information provided by the employed sensors, relevant information about the user behaviour can be drawn. Patterns such as waking up or lunch time, or the identification of the room where the person is expected to be depending on the time, are very useful. This knowledge supports an early identification of changes in behaviour which should be supervised in people suffering mild cognitive impairment. Once the user behavioural patterns have been learnt, the digital assistant can easily provide reminders, not only about medication but also about other daily life activities, such as having a meal or going to bed.

The digital assistant has been, so far, mainly focused on providing social interaction support. The validation of this system has therefore focused on its functionality to start a video call using natural language interaction. Using the smart mirror deployed in a real home setup, during a week different commands were launched and appropriately executed. Video calls were launched in both ways, from the smart mirror to a contact and from a contact to the smart mirror. The digital assistant also provides functionalities for physical activity and medication adherence. In this sense, the use of a calendar with reminders have been implemented. For this functionality, the digital assistant is integrated with the Mi Band 4 smart band. Reminders are sent to both, the smart mirror and the smart band. As such functionality is built upon the Bluetooth communication interface, so far, reminders can only be received in a home environment. Future implementations can also study the use of the smart phone as a bridge to deliver reminders to the smart band.

### 4.3. Use Case 2: Training of Orofacial Musculature

**Figure 29:**
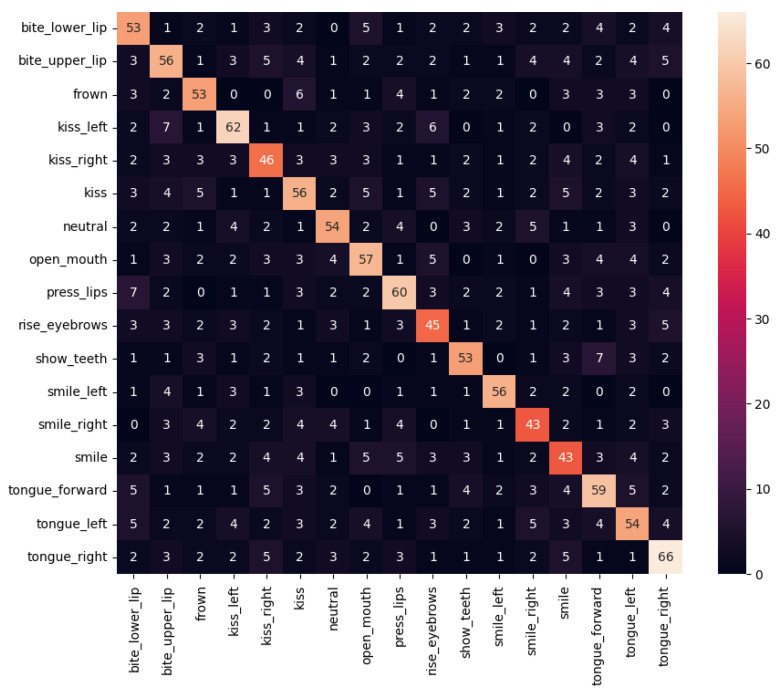
Confusion matrix of the trained orofacial gesture detector.

Figure 13 has shown that the performance of orofacial gestures could be quite subtle. In consequence, as it can be observed in the confusion matrix of the trained model shown in Figure 29, during the execution of these gestures there could be moments in which the appearance between different gestures could be similar. Therefore, users should try to perform gestures with the highest expressiveness possible to avoid this problem. Moreover, the evaluation should consider the time frames with the obtained maximum intensities for each gesture, and ignore the gesture-to-gesture transitions.

### 4.4. Use Case 3: Video-Based Rehabilitation Tool

The success and acceptance of a certain software system is strongly determined by its usability. There are different ways to evaluate the system usability such as the System Usability Scale (SUS) [75] or the Post-Study System Usability Questionnaire (PSSUQ) [76]. Nonetheless, applications specifically designed for older adults, such as those thought to be run in ambient assisted living contexts, call for specific frameworks for usability assessment. This is the case of the International Classification of Functioning, Disability and Health Usability Scale (ICF-US) [77]. This scale offers a way to evaluate digital solutions by focusing the designs on the end-user. This gets the feedback needed to find barriers and enablers in the mock-up phase through validated methodologies and procedures.

The International Classification of Functioning, Disability and Health Usability Scale (ICF-US) [77] has two subscales. The ICF-US I enables a general usability assessment whereas the ICF-US II enables a more detailed assessment, evaluating each component of the design to detect elements that need to be modified (barriers) and good practices (facilitators) to be taken into account for further design of the solution [78,79,80]. Depending on the stage of development of the solution, one or both subscales may be chosen. Thus, ICF-US I can be applied for general usability evaluations, and ICF-US II can be used to evaluate mock-ups in early stages of development to find the weaknesses and strengths of the design. Phyx.io has been evaluated using the ICF-US II scale.

The ICF-US II is comprised of items that identify the components of a digital solution [77]. Each item can be evaluated as a barrier or enabler, with a maximum value of 3 and a minimum value of −3 or as not applicable (N/A) if it does not respond to the item. By doing so, it is possible to identify the components that need to be modified. The ICF-US II is specific to each digital solution although the structure is common and is divided into 3 parts:

1. Components of the application. In the first part we evaluate the components that comprise the digital solution.
2. Detailed usability. This part takes into account the different interaction functionalities as well as the user interface.
3. Overall system evaluation. This last part is composed of a general question about the contact with the system.

The Phyxio evaluation required an evaluator and an observer. The evaluator assesses the rating of each item through observation and interview. Therefore, the evaluator will base his/her answers on the observation of the user’s interaction with the digital solution and on the interview to collect possible reasons why a component is a barrier or to clarify possible doubts about the user’s interaction. On the other hand, the observer collects information considered necessary to ensure redundancy without taking any detail for granted.

The test has been initially prepared and completed by the professionals dedicated to older-adult healthcare. These professionals have a very important role in the Phyxio system, as they are responsible for the management of their patients within the platform. Assigning exercise routines, monitoring the state of health and physical activity or setting appointments or reminders are some of the tasks that this role can perform within the platform. Thus, the evaluation was conducted involving different professional profiles (physiotherapists, therapists) of the nursing home “El Salvador”. A total of 10 participants were selected with an average age of 36.5, maximum 60 years and minimum 25 years. The assessment is carried out in one of the rooms of the physiotherapy team, in their premises.

The evaluator conducting the test introduces the Phyxio platform and provides the user with test credentials for authentication. Then, he/she proposes the healthcare professional to perform the following tasks to evaluate each item of the subscale. The tasks performed by each user are:

- Authenticate on the platform.
- Search something.
- Identify the patients assigned to a professional.
- Explore details of a patient.
- Explore exercises and routines existing in the system.
- Explore exercises and routines assigned to a patient.
- Sort exercises by name and module.
- Create exercises and routines.
- Assign exercises and routines to a patient.
- Delete exercises and routines.
- Delete exercises and routines assigned to a patient.
- Identify the location of the facility to which they belong.
- Edit his/her user details.
- Go to the system start.
- Close session.

Once the task was performed, it can be observed from the results shown in Table 9 that there were some barriers in the application components. 60% of healthcare professionals experienced problems with the top navigation tabs in tasks such as searching for information on an assigned patient, viewing patient routines and exercises, or even not finding the exact functionality of this component. All of these factors complicated the completion of tasks and the navigation within the application. Consequently, 50% had problems with navigation and, more importantly, they found it difficult to understand which profile they were in when they were performing the task. On the other hand, the links provided for ordering exercises and routines were perceived as barriers by 40 percent of users. Tips that were intended to assist users in completing tasks went unnoticed, as 50% did not take them into account. The other evaluated components were not perceived as a barrier, although 20% did not find the use of the drop-down menu useful and were also confused with some of the options displayed in the exercise and routine forms.

Regarding the detailed usability, the biggest barrier encountered by healthcare professionals is the size of the text (60%), especially in links and tips. Furthermore, they considered the text employed for the editing of repetitions and exercise time to be small. For this reason, they also perceived the size of some images (40%) and icons (40%) as a barrier. On the other hand, the used colours and contrast were evaluated positively.

Overall, the evaluation of the application was positive as all users who underwent the test, except for one, were able to understand and perform the tasks they were proposed, although some of them encountered barriers. One user experienced more difficulties, as he found it very difficult to complete certain tasks due to his low level of computer literacy.

### 4.5. Use Case 4: Wearable Motion Monitoring Devices

Regarding the assessment of physical activity monitoring particularly during physical rehabilitation periods, a prototype was tested to collect and analyse data from the Mi Band 4 smart bands worn by six volunteers for a period of approximately five weeks.

The information collected every minute, as mentioned in Section 3.3.3, comprises: the number of steps, the heart rate, the date and time at which the above data was collected and the MAC address corresponding to each smart band, which will be used to identify the band, therefore preserving the identity of the person wearing it. Furthermore, at this stage and as part of the the initial validation, it was also registered the type of walking assistance used by the participants. In this sense, two people can walk without any assistance (Subject E and Subject F), two people walk with the assistance of a walker (Subject B and Subject D), one with crutches (Subject C) and one with a cane (Subject A), which is hold with the same arm that wears the smart band.

**Figure 30:**
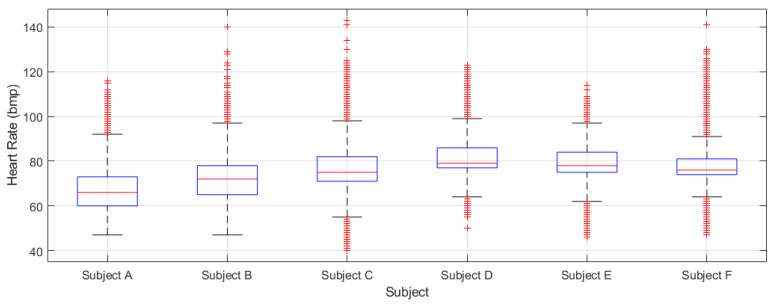
Boxplots of the heart rate registered by the wristbands corresponding to the six participants during the preliminary piloting test.

Figure 30 shows the boxplots obtained from the heart rate data series recorded for each participant during the preliminary prototype tests. These quantitative data will be recorded and analyzed in particular during the physical rehabilitation sessions to monitor the evolution of the participants. It should be noted, that the heart rate is an important indicator of general health and physical condition. Furthermore, it should be mentioned that the heart beat sensor of the Mi Band 4 provided a significant number or saturated non-valid values (approximately 30% of the measurement were not valid). This might have been caused by the recharging periods in which the smartband is not used, and also if the band is not well adjusted and in the right position when wearing it.

**Figure 31:**
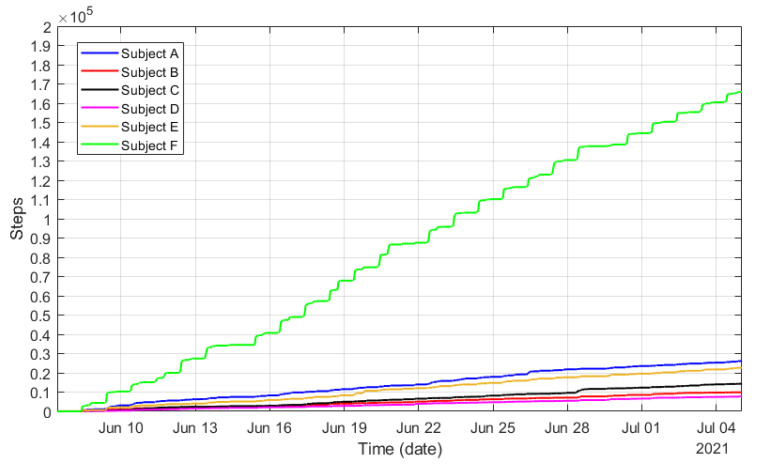
Accumulated steps registered by the wristbands corresponding to the six participants during the preliminary piloting test.

On the other hand, Figure 31 displays the cumulative sum of steps recorded by the smart bands during the preliminary piloting tests. The figure clearly illustrates the level of activity and its trend over a period of time. This quantitative variable will be of great interest to monitor the level of activity during the physical rehabilitation periods.

After an initial analysis of the data, it was also discovered that the Mi Band 4 is not providing and accurate number of steps when using a walking aid. This smart band relies on the swinging movement of the arms when walking to compute the steps walked by a person. Therefore, when the arm holds a cane, for example, it makes it difficult to count the person’s steps and the resulting information is inaccurate. To overcome such limitation, the accuracy and effectiveness of the Mi Band 4 smart band placed in other parts of the body, such as the ankle, is being studied, as well as the use of other devices with better performance in terms of step counting.

## 5. Conclusions

This work presents a smart-mirror based platform, designed fit-for-purpose to support independent living for older adults with neurodegenerative diseases and to promote at-home physical activity and rehabilitation. To this end, a set of hardware and software elements have been proposed and experimentally validated under the use cases designed for two of the SHAPES pilot themes.

One of the main limitations of technological solutions proposed for older adults is the lack of engagement they find or their reluctance to use technology. The proposed platform has focused on overcoming such barriers by proposing a mirror-inspired device, with which older adults are familiar with, as well as by providing natural interfaces (based on voices and the use of a simple RFID band). To this end, both hardware and software systems have gone through a co-design process, therefore ensuring that requirements were well understood and catered for.

The smart mirror concept has gained attention in last few years, particularly as a part of the smart home concept. Nevertheless, its application to the healthy and active ageing paradigm, and more specifically to the two purposes of this work (independent living and at-home physical rehabilitation) is novel and disruptive. In this sense, the main contribution of this paper is the proposal of a smart-mirror-based hardware and software platform, where users and stakeholders have been involved in the design of services to support independent living at home as well as to improve older adults well being. It is also novel the way in which the platform enables a privacy-aware solution, ensuring that data are always under the control of the SHAPES platform. This aspect is achieved while maintaining the functionality levels and cost already achieved by commercial products. It would be totally unfeasible to achieve such functionality levels, aesthetic design and prices, working with non-commercial products. Efforts have therefore been addressed to ensure privacy and data ownership while adopting commercial products (e.g., Mi Band 4, IKEA or Lidl sensors).

The experimental validation of the obtained system has demonstrated the success in meeting the non-functional requirements (privacy, data ownership, performance and accuracy). It has also demonstrated that functional requirements were met, by deploying a real setup and running under different use cases. This has, however, brought to light several limitations of the proposed solutions. The Mi Band 4 smart band performs poorly when the user is using a walking aid. Furthermore, the Phyx.io system has some barriers with regard to the interface design that will be addressed in the following versions.

Finally, as future work, once that the SHAPES smart mirror platform has been validated, the impact of the different interventions it supports will have to be measured. This will be done during the final phase of SHAPES pilots, consisting in the deployment in real-life use cases.

## Figures and Tables

**Table 1:** Review of smart mirror platforms with a potential application in active ageing.

Reference	Type	Field	Features
[19]	Smart mirror	Fitness and health	Kinect camera to capture the user’s movements. The system generates an avatar of the user’s body and a contour shape that indicates the correct exercise position. Interaction with the mirror is through gestures.
[20]	Smart mirror	General	A Raspberry Pi device with a camera and a microphone provide multimedia services while ensuring high-level security throughout the system. Facial recognition system for authentication and voice recognition for interaction. For traffic, news or weather information, Amazon Alexa Voice Service is used.
[21]	Smart mirror	General	A Raspberry Pi device with a camera, a microphone and a speaker offer a smart mirror solution for smart home. Voice recognition and facial recognition techniques for authentication and interaction. Touch interaction through an infrared frame. Automatic wake-up module through an infrared induction module.
[22]	Smart mirror	Healthcare	A Rapsberry Pi device with a camera for facial recognition with a tool based on python, OpenCV and deep learning. Temperature, humidity, pressure, noise and light in the room is measured through a microcontroller with LoRa and Bluetooth wireless transceiver. Mood detection with Microsoft Azure Emotion API, Calendar with Python and CalendarLabs API, Weather with Yahoo Weather API and Location with Google Maps API.
[23]	Smart mirror	General	Smart mirror with virtual assistant to interact with the lighting in the house and provide different information using Alexa. This system is composed of a Raspberry Pi with web cam with microphone and speakers. Motion detection, and face recognition are other characteristics.
[24]	Smart mirror	General	Smart mirror as information panel with clock, date, weather and traffics, alarm clock and daily reminder using Todoist Application and holiday calendar. A Raspberry Pi is the basis of this system.
[25]	Smart mirror	General	Comprises a Raspberry Pi, a display module, a wireless transceiver module, a clock module, a Bluetooth module, a speech synthesis module and auxiliary function module. Information about temperature, weather, date, time, news and other information.
[26]	Smart mirror	Social network	Display with the results of the sentiment analysis using Twitter on Raspberry Pi.
[27]	Smart mirror	Health	This health fitness system has different sensors are used such as DHT11 room temperature sensor, ultrasonic sensor, PIR motion sensor, IR temperature body, a weight foot scale to obtain user data. A USB camera connected to the Raspberry Pi of the smart mirror allows for facial recognition. BIA (Bioelectrical Impedance Analysis) history, BMI (Body Mass Index) analysis, weight history and body temperature are some of the data shown on the display and in the Android Application.
[28]	Smart mirror	General	The functionalities offered are date and time, weather information, personalized news, user’s mail, user’s calendar, music, facial recognition, speech recognition and text-to-speech. An Android application is responsible for mirror access through facial recognition using Microsoft Azure. The information can be obtained by using services such as Dark Sky, RSS of the newspaper Perú 21, Google Calendar and Gmail.
[29]	Smart mirror	Healthcare and psychology	User tracking with respect to the affective state recognition from facial expressions. Ambient light change that gives feedback on the user’s emotional state.
[30]	Smart mirror	Home security	Smart Mirror using DHT 22 sensors and image processing techniques to detect human intrusion like Yolo and Haar cascade classifier of the OpenCV. A Raspberry Pi and a camera to provide the latest news, weather information with touch based control or mobile based control.
[31]	Smart mirror	Home automation	The proposed system has a Raspberry Pi with camera and microphone. An ESP8266 is in charge of the home control. Facial Recognition and Biometric Identification are other functionalities. All the information collected by the mirror can be accessed via web.
[32]	Smart mirror	Smart assistance and General	Through a Raspberry pi and a Camera, facial recognition is performed to load the daily activities that correspond to that identified person. Improvements such as voice control and other functionalities are proposed for the future.
[33]	Smart mirror	General	Project with a Raspberry pi a camera and microcontroller with different sensors like fingerprint sensor, distance sensor, motion sensor, RFID reader and some other components. Protocols such as MQTT and Node-Red are used for the processing and visualisation of sensor data.
[34]	Smart mirror	Health	The proposed smart mirror is composed of a Raspberry Pi, a camera and speakers and a wristband. User detection employs Actcast. The Fitbit APi and wristband are used to acquire the user’s biometrical information.
[35]	Smart mirror	General	Raspberry Pi, 4K LCD Screen and RGB camera on top. The functionalities of the proposed system are portrait log, gesture and speech UI, life rhythm visualisation, touch control module, automatic wake-up module, user registration using Open CV and authentication and emotion detection.
[36]	Smart mirror	Health	Raspberry Pi and web camera for a mirror with facial recognition, emotion recognition and healthcare functionalities. General information like weather, to-do list and clock are the other functionalities of this proposal system. BMI and health data in the mirror using Fitbit app.
[37]	Smart mirror	General	A Rapsberry Pi, microphone, speakers and proximity sensors are the main elements of this design. Interaction through sensors and voice.The different modules it contains are notice, newsfeed, update notification, weather, schedule, status, MQTT events.
[38]	Smart mirror	General	Raspberry Pi, microphone and speakers use ALEXA for voice interaction. The features of this design are date, time, weather, greetings and voice services.
[39]	Smart mirror	Emotional and psychology	A mirror composed of a camera, LED lamps, speakers, microphone and an IoT board. The purposes of this mirrors are the communication with user through a chat bot, estimation of long-term depression through facial recognition, evaluation of conversation mood & tone through speech recognition and behavior identification through pose recognition.
[40]	Smart mirror	General, home automation	A Raspberry Pi, a camera, a microphone and a speaker are included in this mirror. The functionalities offered are face recognition, home automation and voice activation and control subsystem using Amazon services and Alexa skills. It also offers the latest news, calendar, weather forecast, clock, calendar and updates.
[41]	Smart cabinet	Ambient assisted living	This smart cabinet consists of a smart mirror and a medication sensing platform. This solution is defined as Smart Home in a Box (SHIB). Using amazon services, it is intended that this cabinet works as a medication store for the elderly, providing interaction through voice commands.

**Table 2:** Comparison between the services offered by previous related work and the SHAPES smart mirror platform.

		Services of the SHAPES Smart Mirror						
**Field**	**References**	**Call** **Service**	**Fall** **Detector**	**Physical Activity** **Monitor**	**Voice Assistant**	**Calendar** **Reminder**	**Login Service**	**Home** **Monitoring**
General	[20,21,23] [24,25,28] [32,33,35] [37,38,40]	**-**	**-**	**-**	**✓** [20,21,23,25] [28,35,37,38]	**✓** [24]	**✓** [20,21,28,35]	**✓** [33]
Health	[22,27,29] [34,36]	**-**	**-**	**✓** [27,34,36]	**-**	**-**	**✓** [36]	**✓** [22,27]
Fitness	[19]	**-**	**-**	**✓** [19]	**-**	**-**	**-**	**-**
Home automation	[31,40]	**-**	**-**	**-**	**✓** [40]	**-**	**✓** [31]	**-**
Home security	[30]	**-**	**-**	**-**	**-**	**-**	**-**	**-**
Smart assistance	[32]	**-**	**-**	**-**	**-**	**-**	**-**	**-**
Social network	[26]	**-**	**-**	**-**	**-**	**-**	**-**	**-**
Psychology	[29,39]	**-**	**-**	**-**	**✓** [39]	**-**	**-**	**-**
Ambient assisted living	[41]	**-**	**-**	**-**	**✓** [41]	**-**	**-**	**-**

**Table 3:** Summary of services supported by the smart mirror (inputs, outputs and key purpose).

Smart Mirror			
**Service**	**Input**	**Output**	**Purpose**
Home Monitoring	Presence sensors, window and door sensors, temperature and humidity	Periodic activity recognition report	Comfort monitoring Hazard detection Early detection of behavioral changing patterns
Call Service	RFID, microphone, camera	VideoCalls	Easy contact with relatives and healthcare staff using a video-conference system
Fall Detector	IMU sensor	Fall detection alarm	To detect and reduce time for being attended under fall events
Physical Activity Monitor (Phyx.io)	Smartband	Activity Report	To monitor long-term activity
	Depth Camera	Physical Routine feedback	To assist older people in its rehabilitation routine
	Depth Camera	Rehabilitation Report	To assist physiotherapist on patient monitoring
	Depth Camera	Orofacial exercise guide	Orofacial rehabilitation
Voice Assistant	Microphone	Voice interactions	Easy management of smart mirror services
Calendar	Event entries	Reminders (Physical activity, medication and appointments)	To improve adherence to medication and physical activity
Login Service	User credentials, RFID	User sessions log	Grant access to the platform and user profile

**Table 4:** Pilot campaign phases.

Pilot Campaign Phases		
**Part**	**Phase**	**Targets**
Design and preparation	Phase 1: Plan, Design and KPIs	Scenarios to validate initial concepts and approaches
	Phase 2: Mock-up or prototype validation	Prototype/Mock-up validation to assess user acceptance and user experience
	Phase 3: Hands-on experiments	Hand-on Experiments to validate functional elements and gather user feedback
Deployment and execution	Phase 4: Deployment in controlled environment	Experimenting with a single SHAPES digital solution up to demonstrating (part of) the platform in a controlled environment
	Phase 5: Deployment in real-life use cases	Demonstrations in real-life conditions involving the targeted users

**Table 5:** User Requirements for physical activity monitoring with wearable devices.

ID	Requirements
UR-01	USER1: Main persona, older individuals who live alone and wants to keep active.
UR-02	USER2: Therapist or caregiver, the person who supervises the state of the older person.
UR-03	AIM1: Gather information about the physical state of a person measured in terms of his/her activity (number of steps, burnt calories, sleep hours and quality, etc.)
UR-04	AIM2: Track the evolution of such parameters.
UR-05	AIM3: Provide users with feedback about their daily performance in terms of number of steps, burnt calories, slept hours, etc.
UR-06	AIM4: Improve physical condition as result of having a more active life.
UR-07	AIM5: Have the tranquility of having the therapist or caregiver supervising the evolution of the different parameters.
UR-08	HOW1: The system will use a wearable band to track such parameters.
UR-09	HOW2: The system will put all the collected data in a temporal perspective.
UR-10	HOW3: The system will visualize that information using graphics and statistics to help their interpretation.
UR-11	MEASURE1: Is the user more aware about his/her physical activity?
UR-12	GOAL1: Improve the physical activity based on having a more active life.

**Table 6:** User Requirements for orofacial and physical rehabilitation.

ID	Requirement
UR-01	USER1: Main persona, older individuals who are experiencing loss of strength of orofacial or body musculature either due to the degenerative process associated with age or due to an accident or health event such as a stroke.
UR-02	USER2: Therapist, the person that supervises the rehabilitation process.
UR-03	AIM1: Provide a set of scheduled routines, with prescribed exercises that have to be performed following the instructions of an avatar which, when deviations from the baseline exercise occur, will provide instructions so as to correct the postures.
UR-04	AIM2: Track the realization of the routine in order to collect data about performance, time, number of corrections, etc.
UR-05	AIM3: Provide users, therapist and caregivers feedback about the engagement to the rehabilitation plan as well as performance.
UR-06	AIM4: Improve physical condition of the orofacial musculature.
UR-07	AIM5: Feel that the therapist is nearby, supporting the rehabilitation process, in the same way as though the user were at the clinic where the therapist provides support.
UR-08	HOW1: The system will guide the user through the realization of the different routines comprising the rehabilitation plan.
UR-09	HOW2: The system provides easy to interpret graphics and feedback.
UR-10	HOW3: The system provides a video-call system for a direct contact with the therapist when doubts or need for support arise.
UR-11	MEASURE1: Does the user feel his/her physical state or orofacial musculature is improving or maintaining?
UR-12	GOAL1: Improve the physical state or orofacial musculature of the user.

**Table 7:** Communication technologies comparative.

Feature	WiFi	Bluetooth	Z-Wave	ZigBee
Energy consumption	High	10 mW	1 mW	100 mW
Range	1000 m	10 m	30 m	100 m
Cost	Medium	Very low	High	Low
Scalability (number of nodes)	32	20	<6000	6000
Interoperability	WiFi Comptabible devices	Bluethooth compatible devcies	Diferent Manufacturers	Same manufacturer

**Table 8:** Load of the three most highly demanding processes.

Service	Description	CPU	Memory
magic-mirror-2	Service in charge of managing the smart mirror interface	1.08%	8.38%
Miband-dc	Mi Band data collection service	1.34%	3.26%
Fall-detector	Service for data collection and fall detection by means of the MetaMotionR sensor	1.11%	8.79%

**Table 9:** ICF-US II results.

	Barrier	Facilitator	Average [−3,3]
**Components of the application**			
Authentication	0%	100%	2.2
Search Bar	0%	100%	1.7
Dropdown menu	20%	80%	1.2
Top navigation tabs	60%	40%	-0.9
Bottom navigation tabs	0%	100%	1.4
Buttons	0%	100%	1.9
Links	40%	60%	0.5
Forms	20%	80%	1.6
Tips	50%	50%	0.2
Edit form	0%	100%	1.8
Location bar	10%	90%	0.8
Navigation	50%	50%	0.1
**Detailed Usability**			
Image size	40%	60%	0.2
Image color	10%	90%	1.1
Image contrast	0%	100%	1.3
Icons size	40%	60%	0.3
Icons color	0%	100%	1.3
Icons contrast	0%	100%	1.3
Icons intrisic meaning	0%	100%	1.1
Text size	60%	100%	-0.5
Text font	20%	100%	0.6
Text color	0%	100%	1.3
**Overall system evaluation**			
Session flow	10%	90%	1

## Data Availability

Not applicable.
